# S100A4 enhances the pro-fibrotic effect of macrophage to promote collagen capsule formation of *Trichinella spiralis*

**DOI:** 10.1371/journal.pntd.0014647

**Published:** 2026-09-15

**Authors:** Meng Ying Gao, Chen Chen Dong, Yue Chun Tang, Ruo Qi Wang, Li Wang, You Jia Ge, Qiao Yu Geng, Peng Jiang, Zi Fang Zhang

**Affiliations:** Department of Pathogen Biology, School of Basic Medical Sciences, Zhengzhou University, Zhengzhou, People’s Republic of China; University of Buea, CAMEROON

## Abstract

**Background:**

*Trichinella spiralis* is a zoonotic parasitic nematode capable of infecting humans and animals, causing trichinellosis. It poses a critical threat to human health, livestock industry and food safety. The muscle larva of *T. spiralis* is encased within a collagen capsule that is believed to protect it from host immunity. A strong macrophage-dominated inflammatory infiltrate accumulates around the capsule, yet how macrophages contribute to capsule formation remains unclear. Our objective was to investigate the effect of macrophage on collagen capsule formation.

**Methodology/Principal findings:**

The results of immunofluorescence showed that S100A4 ⁺ cells accumulated in *T. spiralis*-infected muscle, and were composed predominantly of CD206 ⁺ macrophages. These macrophages were positioned in direct contact with α-SMA^+^ myofibroblasts. Moreover, numerous cells positive for both CD206 (M2 macrophage canonical marker) and α‑smooth muscle actin (α-SMA, biomarker of myofibroblasts) were observed. Western blot analysis revealed that S100A4 expression was restricted chiefly to M2 macrophage and its expression was markedly amplified by IL-4 together with excretory–secretory (ES) products from muscle larvae of *T. spiralis*. qPCR showed that inhibiting *S100a4* transcription reduced *Arg1* mRNA levels in macrophages activated by ES and IL-4. Immunofluorescence and western blot analysis showed the activated macrophages induced modest upregulation of α-SMA in fibroblasts. Immunohistochemistry revealed that inhibition of S100A4 by niclosamide reduced the expressions of CD206, TGF-β1 and collagen I in *T. spiralis*-infected muscle. Compared with albendazole alone, the combination of niclosamide and albendazole further reduced the larval burden in *T. spiralis*-infected mice.

**Conclusions/Significance:**

In conclusion, infection with *T. spiralis* significantly upregulated S100A4 expression in skeletal muscle-infiltrating macrophages. S100A4 further promoted macrophage M2 polarization. The M2-like macrophages either drove fibroblast activation or acquired myofibroblast-like features, thereby fostering collagen capsule formation. Blocking S100A4 diminished macrophage M2 polarization, prevented collagen capsule formation, and represented a potential adjunctive strategy against trichinellosis.

## Author summary

It is well known that *T. spiralis* provokes a robust inflammatory response encircling the collagen capsule that houses the muscle larva in skeletal muscle. The role of inflammatory cells in collagen capsule formation is debated. In this study, we revealed that macrophages densely accumulated around the muscle larvae of *T. spiralis*. The muscle larvae increased skeletal muscle IL‑4 levels to drive the expression of S100A4 in macrophages. S100A4 further enhanced macrophage M2 polarization. M2-like macrophages induced fibroblast‑to‑myofibroblast transition by secreting S100A4 and TGF-β1, or directly converted into myofibroblast-like cells, which synthesized the extracellular matrix components (ECM) and thereby fostering collagen capsule formation. Inhibition of S100A4 by niclosamide robustly attenuated infection-triggered collagen deposition and concurrently enhanced the efficacy of albendazole against *T. spiralis* muscle larvae.

## Introduction

*Trichinella spiralis* is an intracellular nematode which establishes a chronic infection in skeletal muscle. Infection is usually caused by consumption of raw or improperly cooked meat containing encysted larvae, also referred to as muscle larvae (ML) [1]. Once meat containing encysted larvae is ingested, ML are released after the action of gastric juice. ML invade in the small intestine epithelium, where they develop into adult worms and start to release newborn larvae (NBL). NBL enter into circulation and are carried throughout the body of the host. Only in striated skeletal muscles, the parasite can settle within a single, terminally differentiated skeletal myocyte and develop into ML [2,3]. The skeletal myocyte is transformed into the nurse cell (NC) and ML build an isolated intracellular niche in the nurse cell [4]. The NC-parasite complex is surrounded by a collagenous capsule that provides a suitable long-term parasitic niche for the ML, partially sheltering the larvae from host immune responses and moderately limiting the penetration of anti-*Trichinella* drugs [5].

To survive long-term in the host’s skeletal muscle, *T. spiralis* manipulates the inflammatory response by constantly releasing excretory–secretory (ES) products into the surrounding tissue. ES from the larvae acts as a messenger for communication between the parasite and muscle cell, which plays a key role in development of nurse cell [6]. Studies have suggested that the capsule is composed mainly of type IV and VI collagen, both of which are synthesized by nurse cell [7], while some researchers have speculated that the fibroblasts populating in the infected muscle activated by ES provide the capsular collagen [8]. Some studies have shown that the capsule wall is composed of two layers: the inner layer is of NC origin, and the outer layer is of fibroblast origin [9].

As is well known, the *T. spiralis*-infected muscle exhibits a strong inflammatory reaction around the ML [10]. Previous researchers have observed capsule formation in animals receiving immunosuppressive therapy and therefore speculated that this process might be independent of the host immune response [7,10]*.* For the reason, most studies have focused on the action of *T. spiralis* ES on fibroblasts and muscle cells, ignoring the role of host immune cells in the formation process of collagen capsule [11,12]. To date, very little is known about the effect of inflammatory cells on collagen capsule formation.

The histologic studies of *T. spiralis*-infected muscle showed that macrophages were prominent in inflammatory infiltrates around the nurse cells [13]. Macrophages, an essential component of the innate immunity, are classified into two polarized phenotypes: classically activated M1-like macrophages (pro-inflammatory macrophages that secrete high levels of TNF-α and iNOS) and alternatively activated M2-like macrophages (anti-inflammatory macrophages characterized by elevated expression of Arg-1, CD206 and TGF-β1) [14]. A recent study indicated that extracellular vesicles (EVs) of *T. spiralis* ML might inhibit the synthesis of type I collagen by fibroblasts, while promoting the polarization of macrophages to an M2-like phenotype and high expression of TGF-β [15]. M2-like macrophages are constituting a key driver of fibrotic disease. They can activate fibroblasts by producing pro-fibrotic factors such as TGF-β, and promote ECM deposition [16]. Furthermore, macrophages can transdifferentiate into myofibroblast-like cells in a process termed macrophage–myofibroblast transition (MMT) [17]. Myofibroblasts are the main ECM secretory cells, being considered as a key effector to drive the fibrotic response.

At the protein level, the calcium‑binding protein S100A4 may serve as a key regulator of macrophage M2 polarization, and is widely expressed in various cell types. It plays a crucial role in cancer progression and metastasis [18]. Emerging evidences indicate that S100A4 plays a role in persistent activation of myofibroblasts and is significantly associated with fibrosis [18]. Macrophages are major source of soluble S100A4 in pulmonary fibrosis, and S100A4 released from macrophages is able to enhance the proliferation of fibroblasts [19]. The functional roles of S100A4 in liver fibrosis, systemic sclerosis, and kidney fibrosis, have been established by several investigations [20].

In our previous work, we observed a large number of S100A4^+^ macrophages in *T. spiralis*-infected muscle, which accumulated around the collagen capsule of *T. spiralis*. We hypothesize that S100A4 enhances the pro-fibrotic effect of macrophage to promote collagen capsule formation of *T. spiralis*. In this study, we established a *T. spiralis-*infected mouse model to investigate the dynamic changes and polarization of S100A4^+^ macrophages in *T. spiralis*-infected muscle. *In vitro*, S100A4 expression in ES and IL-4-stimulated macrophages were quantified by western blot. *In vivo*, S100A4 was blocked in *T. spiralis-*infected mice to delineate its influence on macrophage polarization, MMT, collagen deposition and to evaluate the effectiveness of the S100A4 inhibitor against trichinellosis.

## Materials and methods

### Ethics statement

Female BALB/c mice and Kunming mice at 6–8 weeks were obtained from Henan Experimental Animal Center (No. SCXK 2022–0001) and housed in a specific pathogen-free facility. All animal experiments were approved by the Life Science Ethics Committee of Zhengzhou University (No. ZZUIRB GZR 2022–1317).

### Parasite and cell

*T. spiralis* strain (ISS534) was originally isolated from a domestic pig in Henan Province and maintained in our laboratory via serial passaging in Kunming mice.

Murine skeletal muscle fibroblast cell line (NOR-10) was obtained from iCell Bioscience (Shanghai, China) and cultured in DMEM (Servicebio, Wuhan, China) supplemented with 10% fetal bovine serum (FBS, Sangon Biotech, Shanghai, China).

### Preparation of *T. spiralis* muscle larvae ES

ES of *T. spiralis* ML was obtained as previously reported [21]. Muscle larvae were isolated from *T. spiralis*-infected muscles using 1% pepsin- HCl digestion fluid for 3 h at 42°C and washed repeatedly several times in sterile saline. A total of 1.5 × 10⁵ ML were plated in a 10 cm dish with 20 mL RPMI 1640 and incubated at 37°C under 5% CO₂ for 18 h. The supernatant was collected and filtered through a 0.22 μm filter. The filtrate was centrifuged at 4,000 *g*, and the ES of *T. spiralis* ML was obtained. The ES was eliminated endotoxins using the Pierce High Capacity Endotoxin Removal Spin Columns (Thermo Fisher, Waltham, MA, USA). Protein concentrations were determined using a BCA protein assay kit (Solarbio, Beijing, China).

### Synchronous muscle infection with *T. spiralis*

ML were obtained from *T. spiralis*-infected muscles by pepsin-HCl digestion. Fifteen mice were orally infected with 300 *T. spiralis* ML. A single oral dose of pyrantel tartrate (50 mg/kg; MCE, Monmouth Junction, NJ, USA) was administered at 7 days post-infection (dpi) to eliminate intestinal adult worms, thereby establishing a synchronized encystation model of *T. spiralis* muscle larvae [22]. Three mice were euthanized weekly, and diaphragms were harvested for further analysis.

### Peritoneal macrophages isolation and stimulation

Cells from the peritoneal cavity of naive mice were isolated by lavage with 5 mL pre-cooled DMEM. Peritoneal exudate cells were obtained by centrifugation at 500 *g* for 5 min at room temperature and resuspended in DMEM. The cells were seeded in 6-well plates with approximately 4  ×  10^6^ cells/well and cultured in 37°C cell incubator with 5% CO₂. After 4 h, the cells were washed with sterile PBS and the remaining cells were defined as peritoneal macrophages [23]. Subsequently, the macrophages were cultured in DMEM with 20 μg/mL ES, or 20 ng/mL recombinant mouse IL-4 (Novoprotein, Suzhou, China) in 37°C cell incubator with 5% CO₂ for 36 h, respectively. The experimental groups were control (DMEM), ES, ES plus IL-4 and IL-4 alone.

### Co-culture of macrophages with fibroblasts

Peritoneal exudate cells were seeded into 24-well plates at a density of 1 × 10^5^ cells/well. Macrophages were purified by adherence for 4 h at 37°C with 5% CO_2_ and washed with sterile PBS. Then, 20 μg/mL ES and 20 ng/mL IL-4 were added directly to each well with fresh medium. Cells were then incubated for 36 h at 37°C with 5% CO_2_. Macrophages cultured alone were used as the negative control. Subsequently, fibroblasts were added to the macrophage culture system at a ratio of 1:4 (fibroblasts to macrophages) for 48 h at 37°C with 5% CO_2_. Fibroblasts alone and ES‑treated fibroblasts were used as the blank control.

In addition, peritoneal exudate cells were seeded into the upper chamber of the Transwell insert (0.4 μm pore size; BIOFIL, Guangzhou, China) at a density of 4 × 10^6^ cells/well. The lower chamber was filled with 600 μL of DMEM supplemented with 10% FBS. Macrophages were purified by adherence for 4 h at 37°C with 5% CO_2_. The cells were washed with sterile PBS. Then, 20 μg/mL ES and 20 ng/mL IL-4 were added directly to upper and lower chambers with fresh medium. After a 36-hour incubation at 37°C with 5% CO_2_, the upper chambers were inserted into lower chambers. The lower chambers contained 1 × 10⁶ fibroblasts. After a 48-hour incubation at 37°C with 5% CO_2_, the fibroblasts were harvested, and western blot was conducted to determine the levels of α-SMA.

### Targeting S100A4 with niclosamide *in vitro* and *in vivo*

Niclosamide (MCE) was solubilized in DMSO (Solarbio) for *in vitro* experiments. Peritoneal macrophages were seeded into 6-well plates at a density of 5 × 10⁶ cells/well and divided into experimental and control groups. Macrophages in the experimental group were cultured in DMEM supplemented with IL‑4 (20 ng/mL), ES (20 μg/mL), and niclosamide (5 μmol/L) for 36 h. Macrophages in the control group were cultured for 36 h in DMEM supplemented with ES, IL-4, and the same volume of DMSO as the experimental group. Total RNA was extracted from macrophages. Relative mRNA expression levels of *Arg1* and *S100a4* in macrophages were quantified by qPCR.

For the *in vivo* experiments, eighteen *T. spiralis*-infected mice were randomly divided into two groups (n = 9 per group): the control group and the niclosamide treatment group. Mice in niclosamide treatment group were treated with niclosamide (20 mg/kg) via intraperitoneal injections every other day beginning at 10 dpi. Mice in the control group were treated with the appropriate volume of PBS every other day beginning at 10 dpi [24]. Mice were euthanized weekly, and diaphragms were harvested for further analysis.

### Quantitative PCR (qPCR)

Diaphragms were harvested from *T. spiralis*-infected mice at 7, 14 and 21 dpi. Macrophages were stimulated with ES products and IL‑4, with or without niclosamide. Total RNA was extracted from macrophages or diaphragms with Trizol (CWBIO, Jiangsu, China) using a standard protocol, according to the manufacturer’s instruction. RNA was quantified with NanoDrop 2000 (Thermo Fisher). 1 μg of total RNA was reverse-transcribed using a First Strand cDNA Synthesis Kit (Novoprotein). qPCR amplification was performed using the SYBR Green PCR master mix (SparkJade, Shandong, China). *Gapdh* was used as a housekeeping gene, and the relative gene expression was calculated using the 2^-ΔΔCt^ method. Relative mRNA expression levels of *il-4*, *Arg1* and *S100a4* were determined. Each experiment was carried out in triplicate. Primers of the genes used for qPCR in this study are listed in S1 Table.

### Western blot

The macrophages or fibroblasts were washed three times in PBS and lysed by RIPA lysis buffer (Servicebio) at 4°C for 20 min. Sonication was carried out at 10% power for 1 min. Subsequently, the samples were centrifuged at 12,000 *g* and 4°C for 20 min. The supernatants were collected and protein concentrations were determined using a BCA protein assay kit. Samples were boiled for 5 min. After separation by 10% SDS-PAGE, proteins were transferred onto a polyvinylidene difluoride (PVDF; Millipore, Burlington, MA, USA) membrane and the membrane was cut into separate strips. The membranes were blocked with 5% skim milk in TBST at 37°C for 2 h and incubated overnight at 4°C with primary antibodies. The primary antibodies used were as follows: rabbit anti-mouse α-SMA (1:1,000; Abcam, Cambridge, UK), Arg1 (1:1,000; Abcam), iNOS (1:1,000; Abcam) and S100A4 (1:750; Servicebio). iNOS and Arg1 are canonical intracellular functional markers of M1 and M2 polarized macrophages, respectively. After washing with TBST, the membranes were incubated at 37°C for 1 h with HRP-conjugated goat anti-rabbit IgG (1:10,000; Servicebio). Finally, the membranes were colored with an enhanced chemiluminescence kit (Thermo Fisher) and the relative intensities of each band were analyzed using the ImageJ software (NIH, Bethesda, MD, USA). The protein expression levels were standardized to the expression of β-Tubulin or GAPDH. The results were obtained from at least three independent replicates.

### Flow cytometry

Purified peritoneal macrophages were cultured in DMEM with the addition of ES, ES plus IL-4, IL-4 or LPS respectively for 36 h. In subsequent flow cytometric analysis, harvested cells were first gated for leukocytes based on CD45 expression; macrophages were gated as F4/80 ⁺ CD11b⁺ cells using pan-myeloid CD11b and macrophage-restricted F4/80. Fluorescence intensities of CD86 (M1 surface marker) and CD206 (M2 surface marker) within the F4/80 ⁺ CD11b⁺ parent gate was quantified to distinguish the two polarized macrophage subsets. The antibodies used were as follows: FITC anti-mouse CD45 (1:80; Biolegend, San Diego, CA, USA), eFluor 450 anti-mouse F4/80 (1:40; eBioscience, San Diego, CA, USA), PE anti-mouse CD11b (1:80; Biolegend), APC-eFluor 780 anti-mouse CD86 (1:160; eBioscience), and APC anti-mouse CD206 (1:40; Biolegend). Finally, the cells were detected by a full-spectrum flow cytometer (SFLO3; Powclin, Zhejiang, China).

### Therapeutic effect of albendazole and niclosamide combination on *T. spiralis*-infected mice

The *T. spiralis*-infected mice were randomly divided into four groups (n = 4 per group): G1, untreated infected mice; G2, infected mice treated with niclosamide alone; G3, infected mice treated with albendazole (MCE) monotherapy; and G4, infected mice receiving combined albendazole plus niclosamide treatment. Mice in G1 were intraperitoneally injected with PBS every other day starting at 10 dpi; mice in G2 received niclosamide (20 mg/kg) via the same intraperitoneal injection schedule beginning at 10 dpi; mice in G3 were intragastrically administered albendazole (50 mg/kg) once daily for three consecutive days starting at 21 dpi; mice in G4 were given the combined regimen of albendazole and niclosamide following the respective administration routes and timepoints defined for the corresponding monotherapy groups. Mice were euthanized on 35 dpi, and diaphragms were isolated for histopathological analysis. Larvae were isolated from the skeletal muscles of infected mice via digestion to evaluate the effects. Muscle larvae were counted to determine the larval burden, expressed as larvae per gram of muscle. Percentages of larval reduction were expressed as: [(larval burden of control group - larval burden of treatment group) / larval burden of control group] × 100%.

### Histopathological analysis

The diaphragms of mice fixed in 10% neutral-buffered formalin were embedded in paraffin, and sectioned at 3 ~ 5 μm thick. For cultured cells, cells were seeded in 24-well plates and fixed by 4% paraformaldehyde. Muscle sections and cultured cells were blocked with 10% (v/v) normal goat serum (Servicebio) in PBS for 30 min, and incubated with primary antibodies overnight at 4°C. The primary antibodies used in immunohistochemistry (IHC) staining included rabbit anti-mouse F4/80 (1:1,000; Servicebio), anti-mouse CD206 (1:500; Servicebio), anti-mouse S100A4 (1:1,500; Servicebio), anti-mouse TGF-β1(1:200; Servicebio), anti-mouse collagen I (1:500; Servicebio) and anti-mouse α‑SMA (1:1,750; Abcam). Slides were washed three times with PBS and incubated with the secondary antibody HRP goat anti-rabbit IgG (1:500; Servicebio). The primary antibodies used in immunofluorescence (IF) staining included rabbit anti-mouse F4/80 (1:1,000), anti-mouse CD206 (1:1,000), anti-mouse iNOS (1:1,200), anti-mouse S100A4 (1:1,500) and anti-mouse α‑SMA (1:500). The sections were then treated with the TSA plus fluorescence kit (Servicebio) following the standard protocol.

Masson’s trichrome staining was used to assess the collagen deposition in the diaphragms of mice. Following deparaffinization and rehydration, the sections were subjected to Masson’s trichrome staining (Solarbio) in strict accordance with the manufacturer’s instruction.

Results were expressed as the number of positive cells or the positive staining percentage of total area in randomly selected fields (magnification 200×).

### Statistical analysis

Data were expressed as mean ±  SD. Statistical analyses were performed with GraphPad Prism 8.0.2 software (GraphPad, San Diego, CA, USA). Differences between two groups were analyzed using independent-samples *t*-test. Differences between more than two groups were analyzed using one-way ANOVA, two-way ANOVA. *P* value < 0.05 was considered significant.

## Results

### S100A4^+^macrophages were significantly increased in *T. spiralis*-infected muscle

F4/80 is the most commonly used marker for identifying murine macrophages. We first examined the expressions of S100A4 and F4/80 in *T. spiralis-*infected muscle using IF staining and found that the majority of S100A4^+^ cells were also F4/80 positive. The number of S100A4^+^ macrophages increased progressively after 14 dpi (S1A Fig).

**Fig 1:**
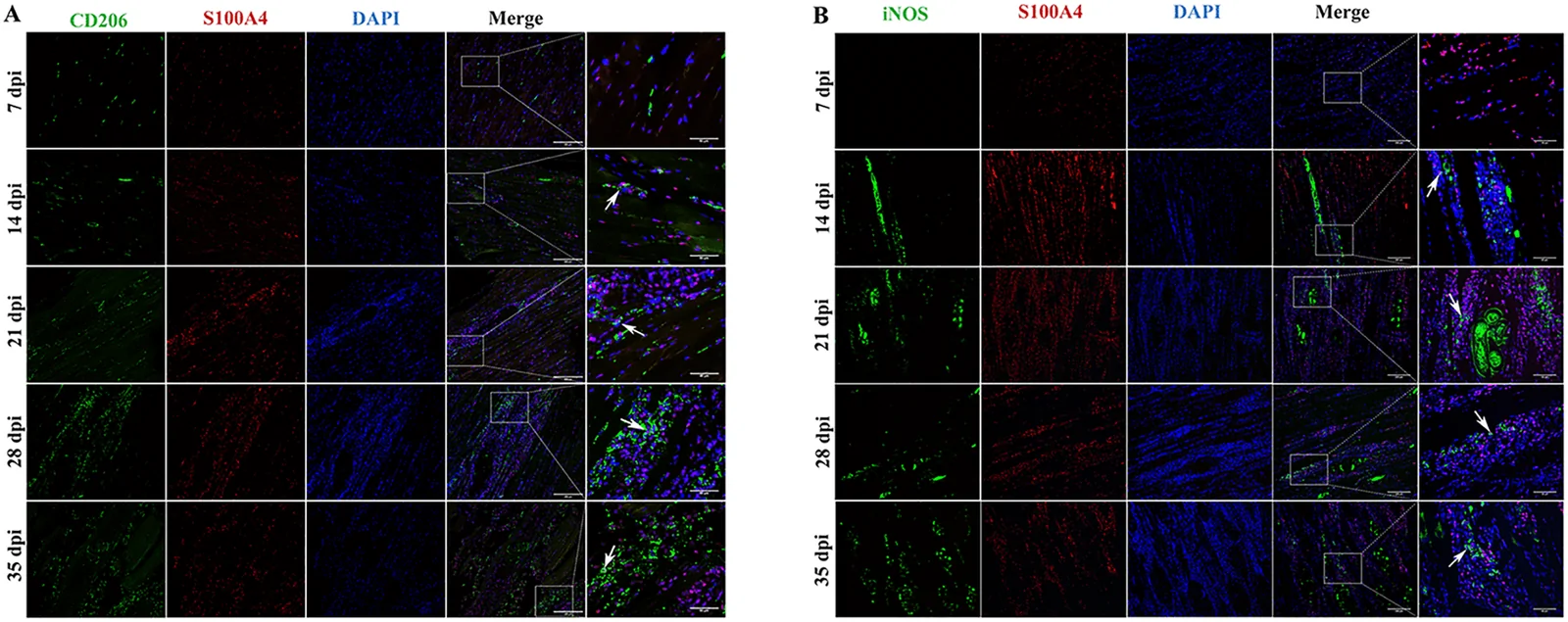
S100A4^+^cells were mainly of CD206^+^macrophages in *T. spiralis*-infected muscle. Diaphragms of *T. spiralis* infected mice were harvested at 7, 14, 21, 28 and 35 dpi. (A) IF staining analyzed CD206 and S100A4 expression in the diaphragms from *T. spiralis*-infected mice. Arrows: CD206^+^S100A4^+^ cells. (B) IF staining analyzed iNOS and S100A4 expression in the diaphragms from *T. spiralis*-infected mice. Arrows: iNOS^+^S100A4^+^ cells. Scale bar, 200 μm (100×), 50 μm (400×).

Furthermore, we used CD206 and iNOS to delineate the polarization of S100A4^+^macrophages from the infected muscle. It is well established that CD206-positive cells are M2 macrophages, whereas iNOS-positive cells represent M1 macrophages. The results showed that both of the CD206^+^S100A4^+^ macrophages (M2-like) and iNOS^+^S100A4^+^ macrophages (M1-like) were increased at 21 dpi. S100A4 was mainly expressed in CD206^+^ macrophages (Fig 1).

In addition, IF staining of the CD206 and α-SMA were conducted. α-SMA is the marker for myofibroblast. The results showed that a large number of α-SMA^+^ myofibroblasts and CD206^+^ macrophages that were in proximity. In addition, IF staining revealed a significant presence of cells co-expressing CD206 and 𝛼-SMA, indicating that macrophages acquired myofibroblast-like features (S1B Fig).

Taken together, these data indicated that increased S100A4^+^ cells in *T. spiralis*-infected muscle were observed, and the CD206^+^ macrophages were the major S100A4 expression cells during the muscle phase of *T. spiralis* infection. CD206^+^ macrophages were direct contact with the α-SMA^+^ myofibroblasts. In addition, CD206^+^ macrophages underwent MMT.

### *T. spiralis* infection induced IL‑4 production in skeletal muscle, which consequently upregulated S100A4 expression in macrophages

Next, we investigated whether *T. spiralis* infection could modulate the expression of S100A4 in macrophages. First, we collected *T. spiralis*-infected muscles and analyzed the gene expression level of *Il-4* by qPCR. The mRNA level of *Il-4* was significantly upregulated in infected muscle at 14 dpi (*P* < 0.01), followed by a gradual decrease (S2 Fig). To mimic the natural microenvironment of *T. spiralis* infection, the macrophages were treated with ES and IL-4.

**Fig 2:**
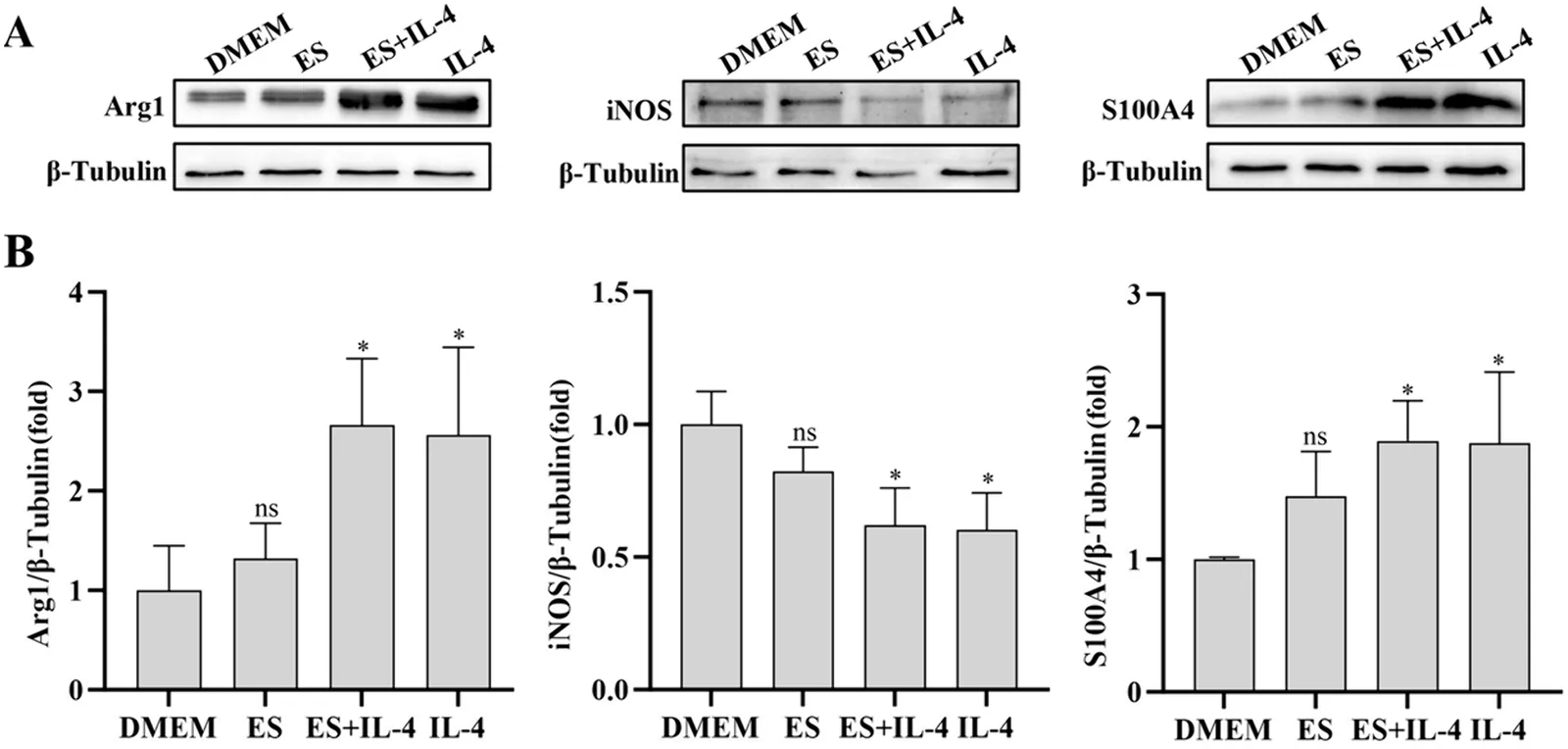
The action of *T. spiralis* muscle larvae ES and IL-4 on macrophage polarization and S100A4 production. Macrophages were derived from peritoneal cavity and were cultured with IL-4 or ES of *T. spiralis* muscle larvae. (A) The protein expressions of Arg1, iNOS and S100A4 in macrophages were analyzed by western blot. (B) The protein expressions were normalized to those of β-Tubulin. DMEM, DMEM treated macrophages; ES, ES (20 μg/mL) treated macrophages; IL-4, IL-4 (20 ng/mL) treated macrophages; ES + IL-4, IL-4 (20 ng/mL) and ES (20 μg/mL) treated macrophages. Results were presented as a mean ± SD of three independent experiments. ^*^P < 0.05. ns, not significant.

Western blot analysis showed that, compared with untreated macrophages, the protein level of Arg1 was significantly increased in IL‑4‑treated (*P* < 0.05) and IL‑4 with ES addition‑treated macrophages (*P* < 0.05), whereas no significant change was observed in ES‑treated macrophages (*P* > 0.05). The protein level of iNOS was significantly reduced in IL‑4‑treated (*P* < 0.05) and IL‑4 with ES addition‑treated macrophages (*P* < 0.05), while no significant difference was found in ES‑treated macrophages (*P* > 0.05). Furthermore, the expression of S100A4 was markedly elevated in IL‑4‑treated (*P* < 0.05) and IL‑4 with ES addition‑treated macrophages (*P* < 0.05). Compared with the control group, the expression of S100A4 exhibited an increasing trend in ES‑treated macrophages, while no significant difference was observed (*P* > 0.05; Fig 2).

Flow cytometric analysis revealed that the percentage of M2 macrophages in IL-4 treated (83.4%) and IL-4 with ES addition treated macrophages (79.1%) was significantly increased compared to untreated macrophages (3.18%) (S3 Fig).

Overall, these results confirmed that S100A4 was predominantly expressed in M2 macrophages, with IL‑4 capable of inducing S100A4 expression independent of *T. spiralis* ML ES products. The proportion of M2 macrophages increased significantly in both IL‑4‑only and IL‑4 plus ES groups. IL‑4 primarily mediated M2 polarization, whereas ES products produced no additional promoting effect.

### The activated macrophages could activate fibroblasts

**Fig 3:**
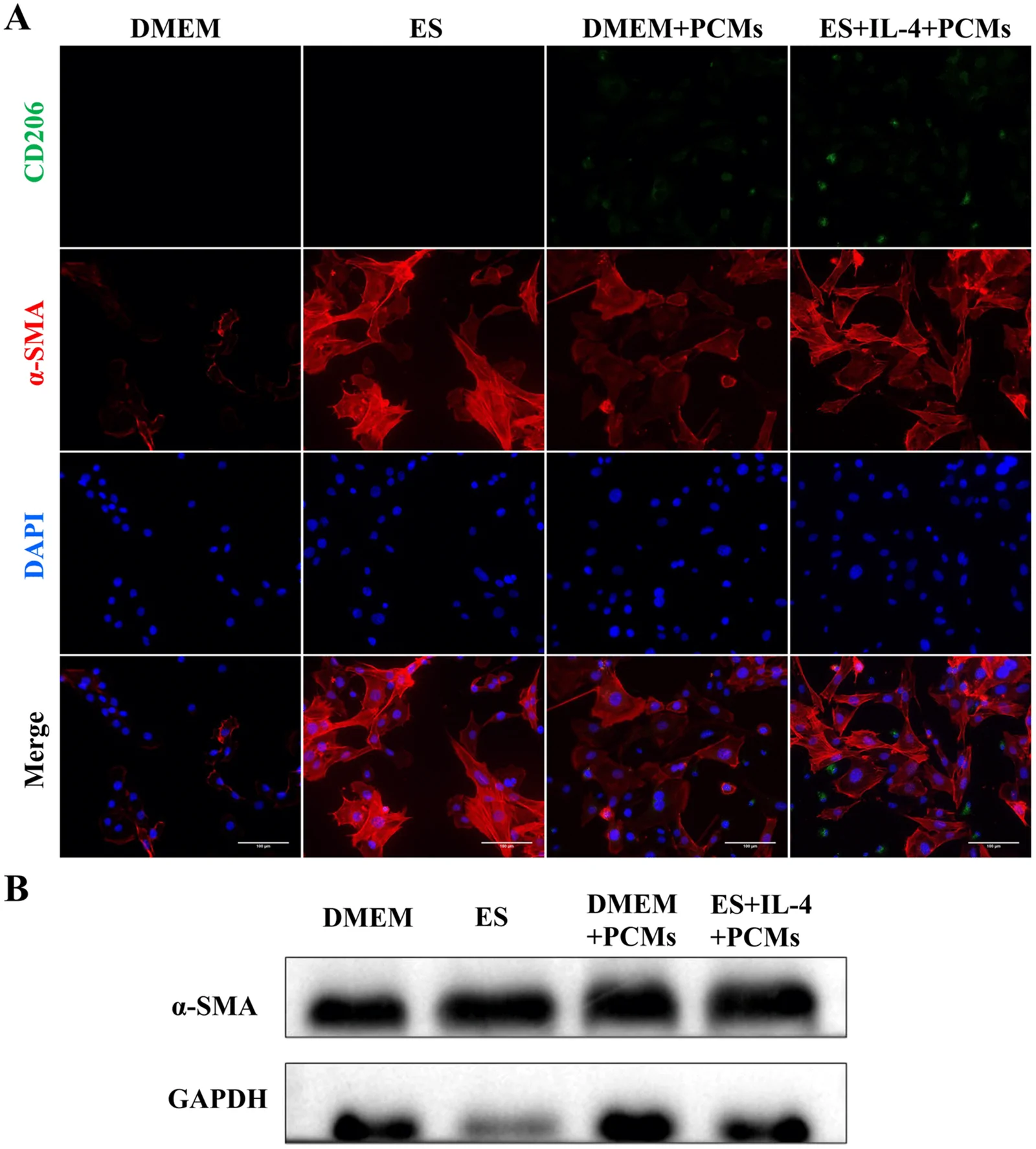
Macrophages stimulated by ES and IL-4 could upregulate α-SMA expression in fibroblasts. Macrophages were treated by ES and IL-4 for 36 h, then the ES and IL-4-stimulated macrophages and naive macrophage were cocultured with fibroblasts for 48 h. DMEM and ES-stimulated fibroblasts as control. (A) The expressions of α-SMA and CD206 were measured by IF. Scale bar, 100 μm. (B) α-SMA expression in fibroblasts was assessed by western blot. PCMs, peritoneal macrophages.

Next, to determine the profibrotic effect of macrophages, macrophages were treated with ES and IL-4, then introduced to the fibroblast cultures. Compared to the fibroblasts only, the expressions of α-SMA were increased in macrophage-fibroblast cocultures. Compared to unactivated macrophages, IL-4 and ES-stimulated macrophages elevated α-SMA expression in co-cultured fibroblasts (Fig 3A). Additionally, ES alone increased α-SMA expression in fibroblasts (Fig 3A). Western blot analysis confirmed that a mild upward trend in α-SMA expression was detected in fibroblasts co-cultured with IL-4 + ES-stimulated macrophages (Fig 3B). The results showed that both activated macrophages and ES of *T. spiralis* muscle larvae had the ability to activate fibroblasts.

### Inhibition S100A4 could diminish macrophage M2 polarization *in vitro*

**Fig 4:**
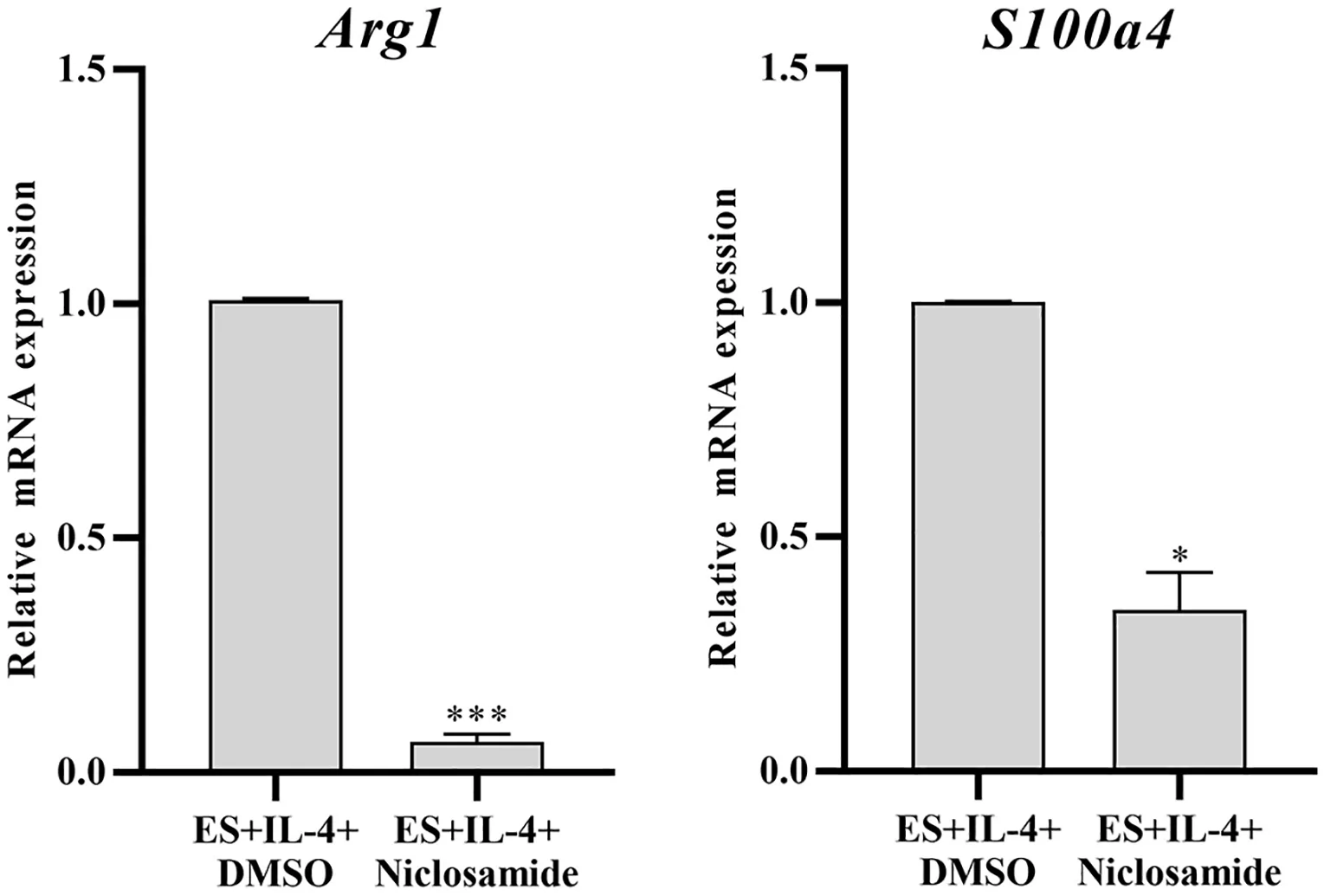
Inhibition of S100A4 diminished macrophage alternative activation. Macrophages were treated by ES + IL-4 + DMSO or with further addition of niclosamide for 36 h, mRNA levels of *Arg1* and *S100a4* in macrophages were analyzed by qPCR. All genes were normalized to *Gapdh*. *P < 0.05, ***P < 0.001.

Niclosamide is the *S100a4* transcriptional inhibitor. To detect the effects of niclosamide on macrophage polarization, ES and IL-4 stimulated macrophages were treated by niclosamide, qPCR was performed to detect the macrophage polarization and the mRNA levels of *S100a4*. Niclosamide treatment significantly decreased the mRNA levels of *S100a4* (*P* < 0.05) and *Arg1* in macrophages (*P* < 0.001) (Fig 4). The results showed that niclosamide could diminish macrophage alternative polarization possibly as a result of the transcriptional suppression of *S100a4*.

### Inhibition of S100A4 could diminish macrophage M2 polarization and attenuate collagen deposition in *T. spiralis*-infected muscle

**Fig 5:**
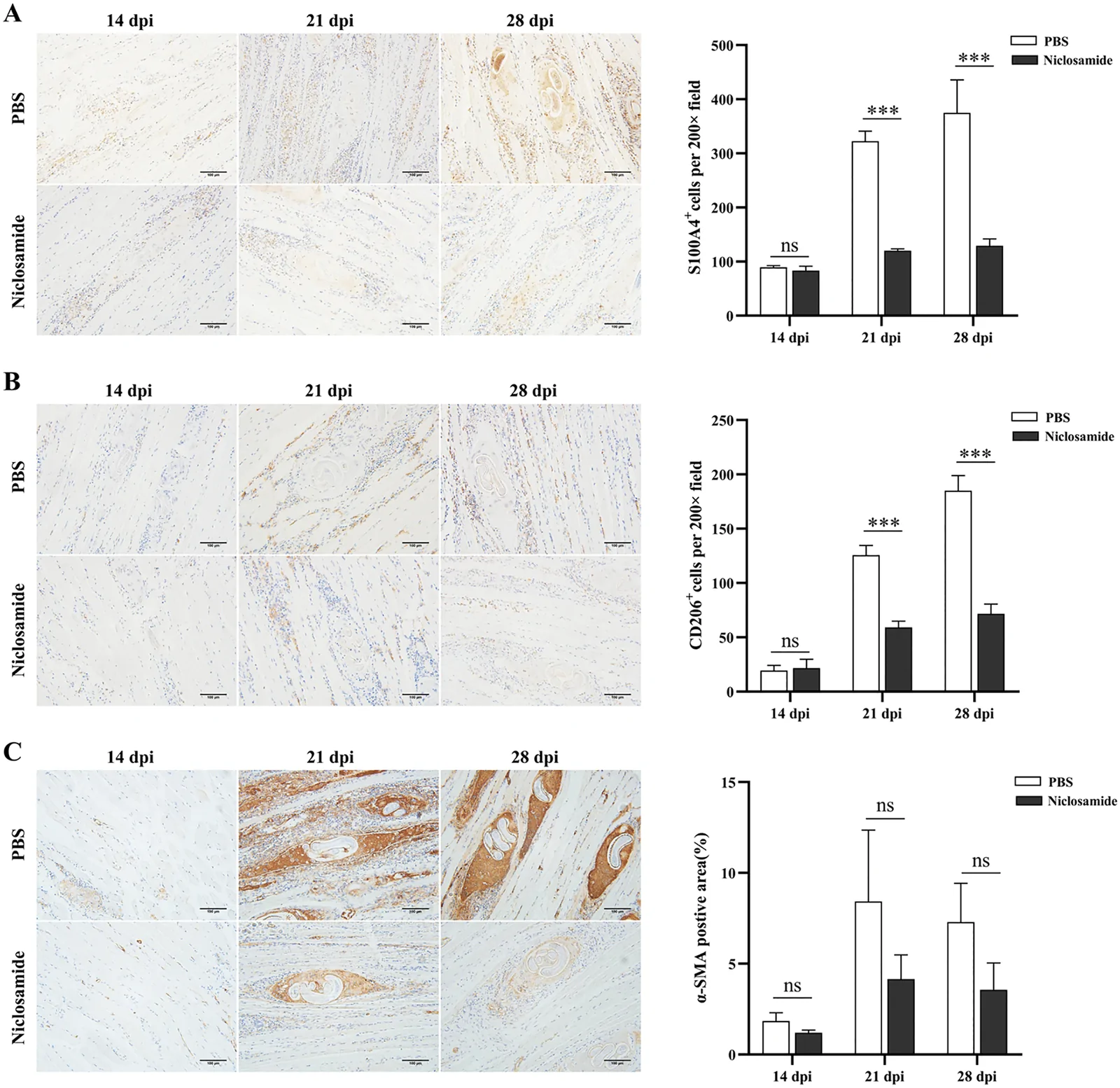
S100A4^+^macrophages could prompt the activation of fibroblasts. *T. spiralis*-infected mice were treated with niclosamide, and the diaphragms were collected at different times after infection. (A) IHC staining of S100A4 in *T. spiralis*-infected diaphragms (n = 3 mice for each time point). (B) IHC staining of CD206 in *T. spiralis*-infected diaphragms (n = 3 mice for each time point). (C) IHC staining of α-SMA in *T. spiralis*-infected diaphragms (n = 3 mice for each time point). Quantifications of S100A4, CD206-positive cells or α-SMA-positive staining area/field were analyzed using ImageJ software. PBS indicated the *T. spiralis*-infected mice were intraperitoneally injected with PBS, while niclosamide indicated the *T. spiralis*-infected mice were intraperitoneally injected with niclosamide. ***P < 0.001. ns, not significant. Scale bar, 100 μm.

To further investigate the role of S100A4^+^ macrophages during collagen capsule formation in *T. spiralis* infection, S100A4 blocked mice were constructed by intraperitoneal injections of niclosamide. S100A4^+^ cells accumulated in *T. spiralis*-infected muscle, while, fewer S100A4^+^ cells were detected in *T. spiralis*-infected muscle treated with niclosamide (*P* < 0.001 at 21dpi, 28 dpi) (Fig 5A). The results suggested that niclosamide successfully reduced the expression of S100A4. The expressions of F4/80 remained essentially unchanged (S4A Fig), while CD206 was statistically down-regulated in *T. spiralis*-infected muscle treated with niclosamide (*P* < 0.001 at 21dpi, 28 dpi) (Fig 5B). Great quantities of α-SMA were observed in *T. spiralis*-infected muscle, while inhibition of S100A4 tended to downregulate α-SMA expression, though no statistically significant differences were detected at 21 and 28 dpi (*P* > 0.05) (Fig 5C). Furthermore, there were significant reductions in the number of CD206^+^α-SMA^+^ cells in niclosamide treated mice (S4B Fig). Together, these data revealed that S100A4 was related to macrophage alternative polarization, and S100A4^+^macrophages could prompt the activation of fibroblasts.

**Fig 6:**
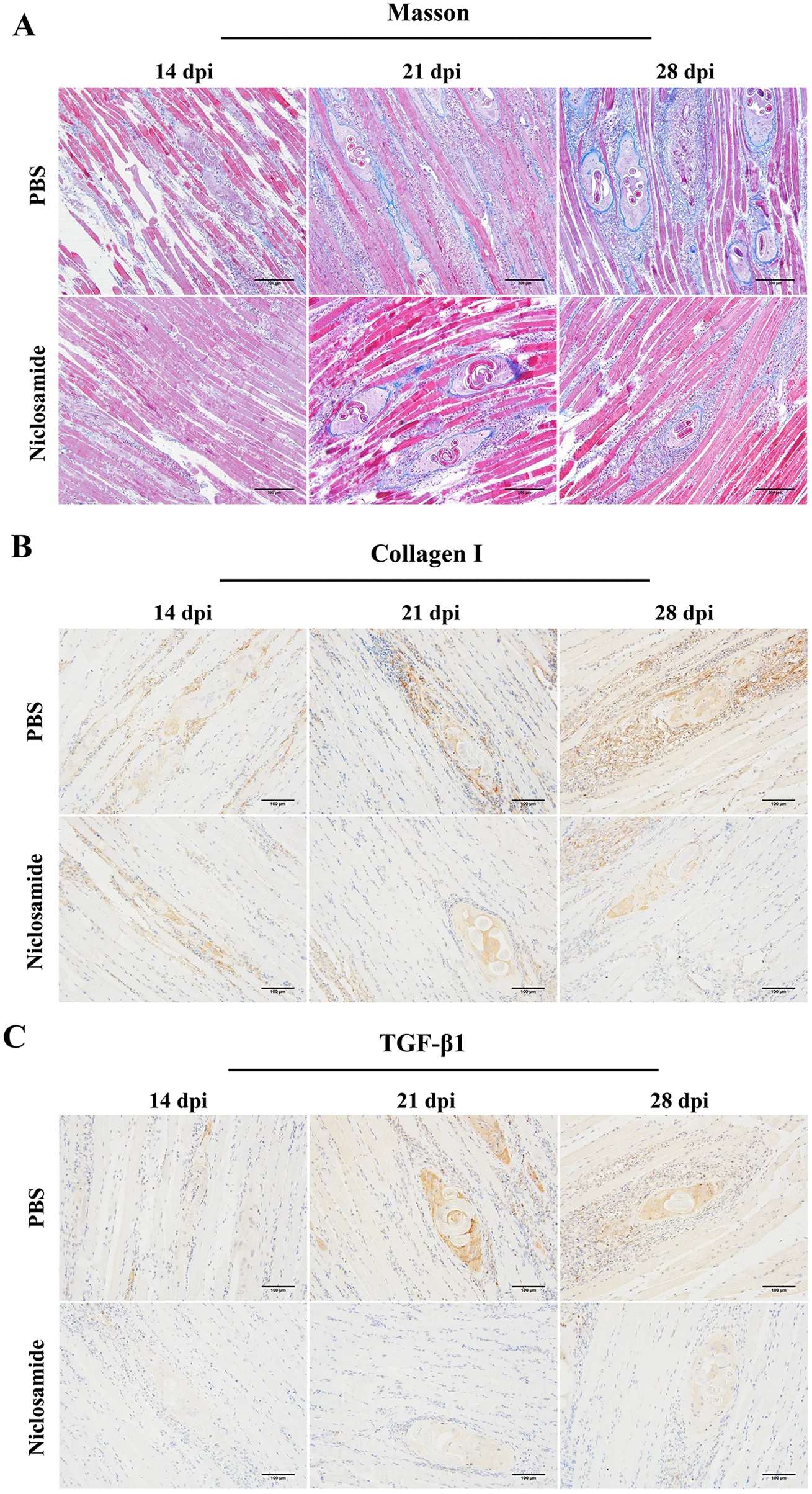
Targeting S100A4 with niclosamide could attenuate TGF-β expression and reduce collagen deposition in *T. spiralis*-infected muscle. *T. spiralis*-infected mice were treated with niclosamide, and the diaphragms were collected at different times after infection. (A) Masson trichrome staining was performed to detect the collagen deposition. (B) IHC staining of collagen type I was performed. (C) IHC staining of TGF-β1 was performed. PBS indicated the *T. spiralis*-infected mice were intraperitoneally injected with PBS, while niclosamide indicated the *T. spiralis*-infected mice were intraperitoneally injected with niclosamide. Scale bar, 100 μm.

To further confirm the above observations, the collagen deposition was examined by Masson trichrome staining. The collagen deposition gradually increased in *T. spiralis*-infected muscle as infection time prolonged. After treatment with niclosamide, the collagen deposition was reduced (Fig 6A). IHC staining of collagen type I was performed, and a similar result was found (Fig 6B). In addition, treatment with niclosamide also reduced the expression of TGF-β1 in *T. spiralis*-infected muscle (Fig 6C).

Altogether, these results revealed that inhibition of S100A4 could inhibit M2 polarization, attenuate TGF-β expression, and block collagen deposition in *T. spiralis*-infected muscle.

### The combined efficacy of niclosamide and albendazole against *T. spiralis* muscular phase infection

**Fig 7:**
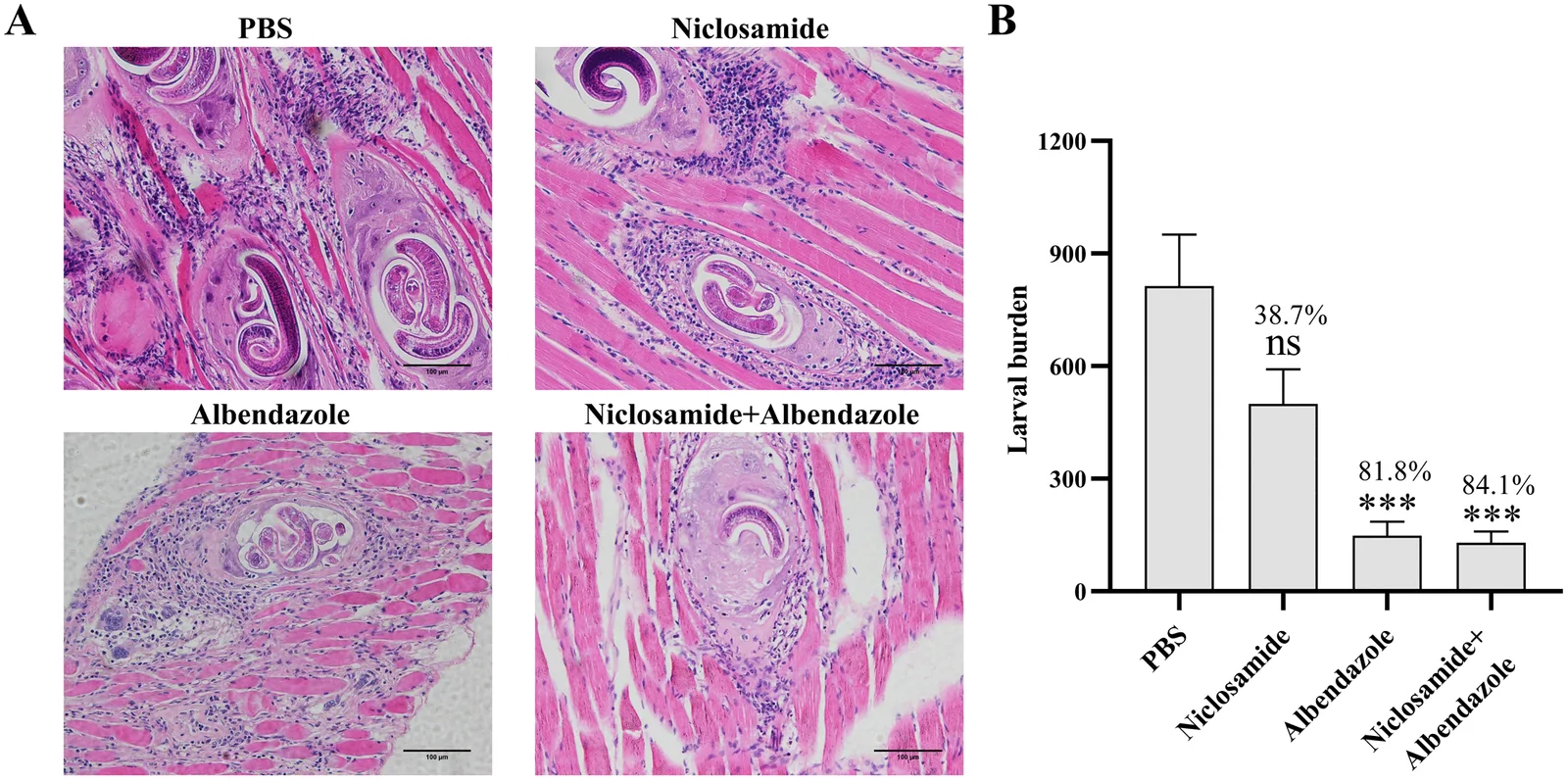
The efficacy of niclosamide and albendazole against *T. spiralis* muscular phase infection. *T. spiralis*-infected mice were treated with albendazole or niclosamide. (A) Representative images of HE staining of *T. spiralis*-infected muscle after treatment. Scale bar, 100 μm. (B) The mean number of larvae per gram (g) of muscle (n = 4 mice per group). PBS indicated the *T. spiralis*-infected mice were intraperitoneally injected with PBS; niclosamide indicated the *T. spiralis*-infected mice were intraperitoneally injected with niclosamide; albendazole indicated the *T. spiralis-*infected mice received albendazole via an oral gavage; niclosamide+albendazole indicated the *T. spiralis*-infected mice were co-treated with albendazole and niclosamide. ***P < 0.001. ns, no significant.

*T. spiralis*-infected mice were treated with albendazole, niclosamide, or albendazole plus niclosamide. On 35 dpi, the therapeutic effect was assessed by comparing muscle larval burden and histopathological examination. HE staining revealed that there was a large number of well-defined cysts in the muscle of infected nontreated group. A massive infiltration of inflammatory cells was existed in the cyst-forming regions. Muscle cells with fragment and necrosis were observed. The muscle larvae reduced in albendazole alone and albendazole-niclosamide combination groups. The inflammatory infiltration was moderate and the cyst wall was slightly thinner in niclosamide alone and albendazole-niclosamide combination groups (Fig 7A).

Compared with nontreated group, the muscle larval burden was significantly reduced in *T. spiralis*-infected mice treated with albendazole (*P* < 0.001), achieved a parasite reduction of 81.8%. Niclosamide treatment alone achieved 38.7% reduction of the larval burden, but there was not statistically significant (*P* > 0.05). The combination of niclosamide and albendazole resulted in an 84.1% reduction of the larval burden (*P* < 0.001), but compared with albendazole alone treated group, there was no statistically significant (*P* > 0.05) (Fig 7B).

## Discussion

In *T. spiralis*-infected muscle, a strong inflammatory reaction is observed around the collagen capsule. Inflammatory infiltrates are primarily composed of macrophages [3]. Macrophages are considered to be the key factor of fibrosis in different organs [14]. However, the role of macrophages in the collagen capsule formation remains unclear. This study provides evidence revealing how S100A4^+^ macrophages drive fibroblast activation, accelerating collagen deposition and thereby promoting collagen capsule formation.

S100A4 has been reported to be involved in the process of fibrosis [25]. In recent study, we noted that S100A4 was strongly upregulated in *T. spiralis*-infected muscle. Macrophages are an important source of soluble S100A4 [26]. We analyzed the co-expression of S100A4 and F4/80 in *T. spiralis*-infected muscle and found that macrophages highly expressed S100A4. Further investigation revealed that S100A4 mainly co-localized with CD206. It has been reported that S100A4 enhances protumor macrophage polarization by control of PPAR-γ-dependent induction of fatty acid oxidation [26]. These suggest that S100A4 may induce macrophages M2 polarization in *T. spiralis*-infected muscle.

M2 macrophages have been shown to promote fibrosis in various organs and tissues [14]. Earlier studies indicated that the activation of fibroblasts by macrophages mainly occurred in close proximity [27]. We performed IF staining and found that a large number of α-SMA^+^ myofibroblasts directly contacted with CD206^+^ macrophages in *T. spiralis*-infected muscle, which indicated that M2 macrophages were crucial for the activation of fibroblasts. Interestingly, the expression of α-SMA in M2 macrophage were significantly higher, which revealed that M2 macrophages underwent MMT. M2 macrophages transform into myofibroblast-like cells that secret excessive ECM and enhance fibrosis [16,17]. These results implicated M2 macrophages as a key player in the collagen capsule formation process of *T. spiralis*.

To clarify the underlying mechanisms of the highly expression of S100A4 in macrophages from *T. spiralis*-infected muscle, we used ES products of *T. spiralis* muscle larvae to stimulate macrophages and analyzed the expression of S100A4 in macrophages. Notably, ES treatment alone exerted a minimal effect on driving M2 polarization. Previous studies have demonstrated a dramatic increase in IL‑4‑producing cells in the diaphragm of *T. spiralis* infected mice [28]. Consistently, we observed markedly elevated IL‑4 levels in *T. spiralis*-infected muscle tissue. Collectively, these findings indicate that macrophages are exposed to dual stimulation by both IL‑4 and ES products within the inflammatory microenvironment established by *T. spiralis* chronic infection. Accordingly, we further investigated the combined effect of ES products and IL‑4 on macrophage M2 polarization *in vitro*.

We found that IL-4 in combination with *T. spiralis* ES products, drove macrophages toward an M2 phenotype and markedly upregulated S100A4 expression. This indicated that the infection of *T. spiralis* established an immune microenvironment characterized by elevated IL-4 expression. Under the combined influence of ES products and IL-4, macrophages underwent M2 polarization and subsequently secreted substantial amounts of S100A4.

Studies have shown that S100A4 is produced and secreted by M2 polarized macrophages in the fibrotic liver and lung. S100A4 derived from macrophages is secreted into the extracellular space and promotes the activation of hepatic stellate cell and fibroblast [19,29]. Based on existing reports and our results, we believe that macrophage-derived S100A4 can activate fibroblasts and contribute to the formation of collagen capsule.

To evaluate the effects of macrophages on fibroblasts, we next co-cultured activated macrophages with fibroblasts. The results confirmed that the IL-4 and ES polarized macrophages induced α-SMA expression in the fibroblasts, and non-polarized macrophages had no significant effect on fibroblast activation. This suggests that M2 macrophages promote fibroblast activation via the secretion of pro-fibrotic factors such as S100A4. We also found that ES from *T. spiralis* muscle larvae could activate fibroblasts, which was consistent with previous studies [30]. These findings indicate that muscle larvae of *T. spiralis* can directly activate fibroblasts through ES products. Furthermore, they may indirectly shape the immune microenvironment by regulating macrophage activation, thereby further promoting fibroblast activation.

Niclosamide is an established anthelminthic drug, and has been proved to be a transcription inhibitor of *S100a4* [24]. Targeting S100A4 has been tried for the treatment of lung fibrosis and successfully ameliorates the development of fibrosis in mouse model [19]. In the *in vitro* study, we found that niclosamide significantly downregulated the mRNA levels of *S100a4* and *Arg1* in macrophages. These showed that inhibiting S100A4 could attenuate macrophage alternative polarization. *In vivo* studies demonstrated that niclosamide was able to decrease the levels of S100A4 in infected muscle, together with a reduction in α-SMA, TGF-β1 and collagen type I, all of which had been demonstrated to be closely related to tissue fibrosis [31]. Furthermore, we found that blocking S100A4 did not influence the quantity of macrophages, but markedly diminished M2 polarization. Consistent with previous research, macrophagic S100A4 enhances macrophage M2-like polarization [26]. Meanwhile, we observed that the skeletal muscle of niclosamide treatment mice had fewer MMT cells compared to the control group, which highlighted S100A4 as a driver for MMT. We have shown here that inhibition of S100A4 could diminish macrophage M2 polarization, reduce the activation of fibroblasts, inhibit MMT, ultimately suppress deposition of collagen.

Niclosamide is approved for treating tapeworm infections. It is not effective against other types of worms [32]. Albendazole is the first-line drug for trichinellosis. The parasiticidal effect of albendazole on encysted larvae of *T. spiralis* was determined in previous studies. Due to differences in the initiation time and frequency of administration, the killing effect differs, generally ranging from 70% to 80% [33–35]. In our study, albendazole monotherapy showed a reduction in encysted larvae with an efficacy of about 81.8%. The combined treatment of albendazole and niclosamide showed a tendency to have better therapeutic efficacy than the single drugs. However, the efficiency of the combined treatment did not show a statistically significant increase. We reasoned that although niclosamide diminished the collagen capsule formation, the capsule structure remained relatively intact, thereby limiting the entry of albendazole. In addition, constrained by the limited number of experimental animals in the present study, we plan to refine the experimental data in future studies by enlarging the sample size.

In this study, we showed that S100A4 was highly upregulated in *T. spiralis*-infected muscle and greater expressed in M2 polarized macrophages. The muscle larvae of *T. spiralis* induced S100A4 expression in macrophages by upregulating IL‑4 levels in the immune microenvironment and through their ES products. S100A4 further promoted macrophage to polarize to M2-like phenotype. The M2-like macrophage promoted fibroblasts transition to myofibroblasts by secreting S100A4 and TGF-β1 or directly transformed into myofibroblast-like cells, which synthesized the ECM and promoted the collagen capsule formation. Niclosamide inhibited S100A4 and attenuated collagen deposition. Though the combination produced a minor numerical improvement in worm burden reduction, niclosamide might weakly augment albendazole’s activity against ML. Further experiments with larger sample sizes are required to validate this tentative trend.

In conclusion, this study demonstrates that S100A4 plays a facilitating effect on macrophages alternative polarization and MMT. Blocking macrophagic S100A4 may develop a novel adjunct therapeutic strategy against trichinellosis. It should be noted, niclosamide is non-specific targeting of S100A4 in macrophages. More experiments specific targeting macrophage-derived S100A4 will be used to strengthen our research results.

## Supporting information

S1 Fig S100A4^+^macrophages increased and transdifferentiated into myofibroblast-like cells in *T. spiralis*-infected muscle. Diaphragms of *T. spiralis*-infected mice were harvested at 7, 14, 21, 28 and 35 dpi. (A) IF staining analyzed F4/80 (green) and S100A4 (red) expressions in the diaphragms from *T. spiralis*-infected mice. Arrows: F4/80^+^S100A4^+^ cells. (B) IF staining analyzed CD206 (green) and 𝛼-SMA (red) expression in the diaphragms from *T. spiralis*-infected mice. Yellow arrows: CD206^+^𝛼-SMA^+^ cells. Scale bars, 200 μm, 100 μm.
(TIF)

S2 Fig The mRNA levels of *Il-4* in *T. spiralis*-infected muscle. qPCR was used to detect the gene expression of *Il-4* in *T. spiralis*-infected muscle at different times on 7, 14 and 21 dpi (n = 3 mice for each time point). The mRNA levels of *Il-4* were expressed as relative expression to *Gapdh*. **P < 0.01. ns, not significant.
(TIF)

S3 Fig The action of *T. spiralis* muscle larvae ES and IL-4 on macrophage polarization. The purified peritoneal macrophages were cultured in DMEM with the addition of ES, ES + IL-4, IL-4 or LPS respectively for 36 h. Flow cytometric analysis of CD206 and CD86 expression on macrophages and the proportion of CD206 and CD86 positive macrophages.
(TIF)

S4 Fig S100A4 blockade inhibited MMT. *T. spiralis*-infected mice were treated with niclosamide, and the diaphragms were collected at different times after infection. (A) IHC staining of F4/80 in *T. spiralis*-infected diaphragms (n = 3 mice for each time point). Scale bar, 100 μm. Quantification of F4/80 positive staining cells was analyzed using ImageJ software. (B) IF staining analyzed CD206 and α-SMA expression in the diaphragms from *T. spiralis*-infected mice. PBS indicated the *T. spiralis*-infected mice were intraperitoneally injected with PBS, while niclosamide indicated the *T. spiralis*-infected mice were intraperitoneally injected with niclosamide. ns, not significant. Scale bar, 200 μm (100×), 100 μm (200×).
(TIF)

S1 File Raw images of western blot. (TIF)

S1 Table Primer sequences of *Il-4*, *Arg1* and *S100a4* in qPCR assays. (DOCX)

## References

[1] Rostami A, Gamble HR, Dupouy-Camet J, Khazan H, Bruschi F. Meat sources of infection for outbreaks of human trichinellosis. Food Microbiol. 2017;64:65–71. doi: 10.1016/j.fm.2016.12.012 28213036

[2] Zarlenga D, Thompson P, Pozio E. *Trichinella* species and genotypes. Res Vet Sci. 2020;133:289–96. doi: 10.1016/j.rvsc.2020.08.012 33199264

[3] Sun Q, Huang J, Gu Y, Liu S, Zhu X. Dynamic changes of macrophage activation in mice infected with *Trichinella spiralis*. Int Immunopharmacol. 2022;108:108716. doi: 10.1016/j.intimp.2022.108716 35344812

[4] Liu X, Zhang B, Zhang Z, Wang X, Zhang T, Huang H, et al. Interleukin-13 partly induced by the NLRP3 inflammasome promotes *Trichinella spiralis* encapsulation in infected mice. Vet Parasitol. 2025;334:110386. doi: 10.1016/j.vetpar.2024.110386 39732108

[5] Rayia DA, Othman A, Harras S, Helal D, Dawood L, Soliman S. Bevacizumab: a new take on therapy of muscle phase of *Trichinella spiralis* infection. Acta Trop. 2022;230:106409. doi: 10.1016/j.actatropica.2022.106409 35300938

[6] Wu Z, Nagano I, Takahashi Y. *Trichinella*: what is going on during nurse cell formation?. Vet Parasitol. 2013;194(2–4):155–9. doi: 10.1016/j.vetpar.2013.01.044 23433992

[7] Polvere RI, Kabbash CA, Capó VA, Kadan I, Despommier DD. *Trichinella spiralis*: synthesis of type IV and type VI collagen during nurse cell formation. Exp Parasitol. 1997;86(3):191–9. doi: 10.1006/expr.1997.4180 9225769

[8] Haehling E, Niederkorn JY, Stewart GL. *Trichinella spiralis* and *Trichinella pseudospiralis* induce collagen synthesis by host fibroblasts in vitro and in vivo. Int J Parasitol. 1995;25(12):1393–400. doi: 10.1016/0020-7519(95)00080-1 8719950

[9] Wu Z, Sofronic-Milosavljevic L, Nagano I, Takahashi Y. *Trichinella spiralis*: nurse cell formation with emphasis on analogy to muscle cell repair. Parasit Vectors. 2008;1(1):27. doi: 10.1186/1756-3305-1-27 18710582 PMC2538513

[10] Vega-Sánchez V, Gómez-De-Anda F-R, Calderón-Domínguez G, Ramírez-Y-Ramírez M-C-D-S, Reyes-Rodríguez N-E, Zepeda-Velázquez A-P, et al. Kinetics of eosinophils during development of the cellular infiltrate surrounding the nurse cell of *Trichinella spiralis* in experimentally infected mice. Pathogens. 2021;10(11):1382. doi: 10.3390/pathogens10111382 34832538 PMC8617616

[11] Hernández-Ancheyta L, Salinas-Tobón MDR, Cifuentes-Goches JC, Hernández-Sánchez J. *Trichinella spiralis* muscle larvae excretory-secretory products induce changes in cytoskeletal and myogenic transcription factors in primary myoblast cultures. Int J Parasitol. 2018;48(3–4):275–85. doi: 10.1016/j.ijpara.2017.10.002 29258830

[12] Park MK, Cho MK, Kang SA, Kim BY, Yu HS. The induction of the collagen capsule synthesis by *Trichinella spiralis* is closely related to protease-activated receptor 2. Vet Parasitol. 2016;230:56–61. doi: 10.1016/j.vetpar.2016.11.001 27884442

[13] Beiting DP, Bliss SK, Schlafer DH, Roberts VL, Appleton JA. Interleukin-10 limits local and body cavity inflammation during infection with muscle-stage *Trichinella spiralis*. Infect Immun. 2004;72(6):3129–37. doi: 10.1128/IAI.72.6.3129-3137.2004 15155614 PMC415664

[14] Jiang Y, Cai R, Huang Y, Zhu L, Xiao L, Wang C, et al. Macrophages in organ fibrosis: from pathogenesis to therapeutic targets. Cell Death Discov. 2024;10(1):487. doi: 10.1038/s41420-024-02247-1 39632841 PMC11618518

[15] Wu J, Liao Y, Li D, Zhu Z, Zhang L, Wu Z, et al. Extracellular vesicles derived from *Trichinella Spiralis* larvae promote the polarization of macrophages to M2b type and inhibit the activation of fibroblasts. Front Immunol. 2022;13:974332. doi: 10.3389/fimmu.2022.974332 36211336 PMC9532625

[16] Tung NTC, Nogami M, Iwasaki M, Yahara Y, Seki S, Makino H, et al. M2-like macrophages derived from THP-1 cells promote myofibroblast differentiation of synovial fibroblasts in association with the TGF-β1/SMAD2/3 signaling pathway. Sci Rep. 2025;15(1):25505. doi: 10.1038/s41598-025-10858-6 40665033 PMC12264265

[17] Li X, Liu Y, Tang Y, Xia Z. Transformation of macrophages into myofibroblasts in fibrosis-related diseases: emerging biological concepts and potential mechanism. Front Immunol. 2024;15:1474688. doi: 10.3389/fimmu.2024.1474688 39386212 PMC11461261

[18] Fei F, Qu J, Li C, Wang X, Li Y, Zhang S. Role of metastasis-induced protein S100A4 in human non-tumor pathophysiologies. Cell Biosci. 2017;7:64. doi: 10.1186/s13578-017-0191-1 29204268 PMC5702147

[19] Zhang W, Ohno S, Steer B, Klee S, Staab-Weijnitz CA, Wagner D, et al. S100a4 is secreted by alternatively activated alveolar macrophages and promotes activation of lung fibroblasts in pulmonary fibrosis. Front Immunol. 2018;9:1216. doi: 10.3389/fimmu.2018.01216 29910813 PMC5992816

[20] O’Reilly S. S100A4 a classical DAMP as a therapeutic target in fibrosis. Matrix Biol. 2024;127:1–7. doi: 10.1016/j.matbio.2024.01.002 38219976

[21] Xu Q, Lin X, Song L, Ren Y, Bai X, Zhao X, et al. *Trichinella spiralis* excretory-secretory protein alleviates autoimmune thyroiditis by modulating Th17/Treg balance via the STAT3/STAT5 pathway. Acta Trop. 2025;268:107706. doi: 10.1016/j.actatropica.2025.107706 40562184

[22] Howes HL Jr, Lynch JE. Anthelmintic studies with pyrantel. I. Therapeutic and prophylactic efficacy against the enteral stages of various helminths in mice and dogs. J Parasitol. 1967;53(5):1085–91. doi: 10.2307/3276845 6062062

[23] Knuth A-K, Huard A, Naeem Z, Rappl P, Bauer R, Mota AC, et al. Apoptotic cells induce proliferation of peritoneal macrophages. Int J Mol Sci. 2021;22(5):2230. doi: 10.3390/ijms22052230 33668084 PMC7956251

[24] Milani M, Mammarella E, Rossi S, Miele C, Lattante S, Sabatelli M, et al. Targeting S100A4 with niclosamide attenuates inflammatory and profibrotic pathways in models of amyotrophic lateral sclerosis. J Neuroinflammation. 2021;18(1):132. doi: 10.1186/s12974-021-02184-1 34118929 PMC8196441

[25] Zhang C, Bai K, Li D. Downregulation of S100 calcium-binding A4 (S100A4) ameliorates hepatic fibrosis via regulating Wnt/β-catenin signaling pathway. Eur J Histochem. 2025;69(2):4186. doi: 10.4081/ejh.2025.4186 40223805 PMC12051413

[26] Liu S, Zhang H, Li Y, Zhang Y, Bian Y, Zeng Y, et al. S100A4 enhances protumor macrophage polarization by control of PPAR-γ-dependent induction of fatty acid oxidation. J Immunother Cancer. 2021;9(6):e002548. doi: 10.1136/jitc-2021-002548 34145030 PMC8215236

[27] Xu Y, Ying L, Lang JK, Hinz B, Zhao R. Modeling mechanical activation of macrophages during pulmonary fibrogenesis for targeted anti-fibrosis therapy. Sci Adv. 2024;10(13):eadj9559. doi: 10.1126/sciadv.adj9559 38552026 PMC10980276

[28] Fabre MV, Beiting DP, Bliss SK, Appleton JA. Immunity to *Trichinella spiralis* muscle infection. Vet Parasitol. 2009;159(3–4):245–8. doi: 10.1016/j.vetpar.2008.10.051 19070961 PMC3449155

[29] Chen L, Li J, Zhang J, Dai C, Liu X, Wang J, et al. S100A4 promotes liver fibrosis via activation of hepatic stellate cells. J Hepatol. 2015;62(1):156–64. doi: 10.1016/j.jhep.2014.07.035 25111176

[30] Park MK, Kim H-J, Cho MK, Kang SA, Park SY, Jang SB, et al. Identification of a host collagen inducing factor from the excretory secretory proteins of *Trichinella spiralis*. PLoS Negl Trop Dis. 2018;12(11):e0006516. doi: 10.1371/journal.pntd.0006516 30383752 PMC6233931

[31] Meng X-M, Nikolic-Paterson DJ, Lan HY. TGF-β: the master regulator of fibrosis. Nat Rev Nephrol. 2016;12(6):325–38. doi: 10.1038/nrneph.2016.48 27108839

[32] Al-Kuraishy HM, Al-Gareeb AI, Alzahrani KJ, Alexiou A, Batiha GE-S. Niclosamide for Covid-19: bridging the gap. Mol Biol Rep. 2021;48(12):8195–202. doi: 10.1007/s11033-021-06770-7 34664162 PMC8522539

[33] Fahmy AM, Diab TM. Therapeutic efficacy of albendazole and mefloquine alone or in combination against early and late stages of *Trichinella Spiralis* infection in mice. Helminthologia. 2021;58(2):179–87. doi: 10.2478/helm-2021-0016 34248378 PMC8256455

[34] Ibrahim AF, Selim SM, Shafey DA, Sweed DM, Farag SA, Gouda MA. Appraisal of chitosan-coated lipid nano-combination with miltefosine and albendazole in the treatment of murine trichinellosis: experimental study with evaluation of immunological and immunohistochemical parameters. Acta Parasitol. 2024;69(1):929–50. doi: 10.1007/s11686-024-00799-x 38489009 PMC11001732

[35] El-Wakil ES, Khodear GAM, Ahmed HES, Ibrahim GIK, Hegab F, Abdo SM. Therapeutic efficacy of albendazole and berberine loaded on bovine serum albumin nanoparticles on intestinal and muscular phases of experimental trichinellosis. Acta Trop. 2023;241:106896. doi: 10.1016/j.actatropica.2023.106896 36921748

