# Supplementary material for: S100A4 enhances the pro-fibrotic effect of macrophage to promote collagen capsule formation of Trichinella spiralis
Source: PLoS Negl Trop Dis. 2026 Sep 15;20(9):e0014647. doi: 10.1371/journal.pntd.0014647 (PMC13577483; doi:10.1371/journal.pntd.0014647)
Supplement: Supplementary file 1 [file pntd.0014647.s006.docx]

S1 Table. Primer sequences of *Il-4*, *Arg1* and *S100a4* in qPCR assays.

| Genes | Sequences (5ʹ to 3ʹ) | GenBank no. |
| --- | --- | --- |
| *Il-4* | F: CATCCTGCTCTTCTTTCTCG  R: CCCTTCTCCTGTGACCTCG | NM_021283.4 |
| *Arg1* | F: GGGGAAAGCCAATGAAG  R: TGGTTGTCAGGGGAGTGT | NM_007482.3 |
| *S100a4* | F: TCAGCACTTCCTCTCTCTTGG  R: AACTTGTCACCCTCTTTGCC | NM_011311.2 |
| *Gapdh* | F: TACCCCCAATGTGTCCGTC  R: AAGAGTGGGAGTTGCTGTTGA | NM_008084.6 |
