# Supplementary material for: S100A4 enhances the pro-fibrotic effect of macrophage to promote collagen capsule formation of Trichinella spiralis
Source: PLoS Negl Trop Dis. 2026 Sep 15;20(9):e0014647. doi: 10.1371/journal.pntd.0014647 (PMC13577483; doi:10.1371/journal.pntd.0014647)
Supplement: Supplementary file 2 [file pntd.0014647.s007.docx]

**> Reviewer's Responses to Questions**

**Methods**

Reviewer #1: All the method sections need more detail including but not only the suppliers used for the consumables, the conditions used and numbers of animals per group. Methods should be written so that the reader can recreate the protocol if needed. Some detail below -but every section needs editing.

Response: Thank you for your comments and suggestions. In the method section, we have added more detail.

Line 136 – why only female mice?

Response: Thank you for your comments. There is no strict gender restriction for establishing *T. spiralis*-infected animal model. Both female and male mice have been widely used in existing studies. In addition, female mice are relatively docile and easier to handle during experimental operations. Therefore, only female mice were selected in this study.

Line 139 – include ethics number details

Response: Thank you for your comments and suggestions. We have added ethics number details in the revised manuscript (at line 144).

Section at line 146 - what temperature were the ML cultured at? How many ML in what volume?

Response: Thank you for your comments and suggestions. We have added relevant information in the revised manuscript (at lines 156-157).

Section at line 156 -number of mice in each group? ML used for infections - were these the ML that were cultured or just recovered from the muscle tissue and washed?

Response: Thank you for your comments and suggestions. We have added relevant information in the revised manuscript (at lines 165-170).

Section at line 163 – peritoneal cavity of which mice? Peritoneal macrophages were those stuck to the plate? More detail needed. Supplier details needed for IL-4. Details for cell stimulation – what temperature? Details for Western blot.

Response: Thank you for your comments. Sorry for not describing clearly in the section of peritoneal macrophages isolation and stimulation. We harvested peritoneal cells from uninfected mice. According to relevant references (Zhu J, et al. Parasitic antigens alter macrophage polarization during *Schistosoma japonicum* infection in mice. Parasit Vectors. 2014 7:122; Knuth AK, et al. Apoptotic cells induce proliferation of peritoneal macrophages. Int J Mol Sci. 2021; 22(5): 2230.), the collected peritoneal cells were cultured for 3–4 h to allow adherence, and flow cytometric analysis showed that their purity reached 98%. Details of cell stimulation, incubation temperature (at lines 175-184), and Western blot procedures (at lines 240-260) have been supplemented in the revised manuscript.

Section at line 173 – details of culture, immunofluorescence and Western blot.

Response: Thank you for your comments and suggestions. We have added relevant information in the revised manuscript (at lines186-205, 240-260, 300-303).

Section at line 181 – specific details for RNA extraction and qPCR including supplier names, gene accession numbers

Response: Thank you for your comments and suggestions. We have added relevant information in the revised manuscript (S1 Table).

Section at line 187 – details needed

Response: Thank you for your comments and suggestions. We have added relevant information in the revised manuscript (at lines at lines 240-260).

**Results**

Reviewer #1: The results are difficult to follow at times, particularly as enough information hasn't been provided regarding the cell markers being used, specifically for non-immunology specialists that may be reading this manuscript.

Response: Thank you for your comments and suggestions. We have supplemented the introduction of cell markers in the revised manuscript.

Line 258 – what is F4/80?

Response: Thank you for your comments and suggestions. F4/80 is a well-characterized cell surface glycoprotein and the most commonly used marker for identifying murine macrophages. It is frequently employed in immunohistochemistry and immunofluorescence to label macrophages, and is often combined with additional markers such as iNOS or CD206 to distinguish macrophage subsets. We have added relevant information in the revised manuscript (Ou DL, et al. Nano-modified viruses prime the tumor microenvironment and promote the photodynamic virotherapy in liver cancer. J Biomed Sci 2024 Jan 02;31(1):1) (at lines 320-321, 325-326).

Line 292 – why was only IL4 analysed?

Response: Thank you for your comments. We attempted to induce M2 polarization of macrophages using excretory-secretory (ES) products from *T. spiralis* muscle larvae. Notably, ES treatment alone exerted a minimal effect on driving M2 polarization. Previous studies have demonstrated a dramatic increase in IL‑4‑producing cells in the diaphragm of *T. spiralis* infected mice. Consistently, we observed markedly elevated IL‑4 levels in *T. spiralis*-infected muscle. Collectively, these findings indicate that macrophages are exposed to dual stimulation by both IL‑4 and ES products within the inflammatory microenvironment established by *T. spiralis* chronic infection. Accordingly, we further investigated the combined effect of ES products and IL‑4 on macrophage M2 polarization in vitro. A detailed explanation has been provided in the discussion section (lines 544-556).

Line 393 – Masson

Response: Thank you for your comments and suggestions. We have made revision according to the reviewer’ suggestion (line 458).

Figure S3 – how have the CD206 and F4/80 panels been gated? The ES+IL4 and IL4 panels need further confidence.

Response: Thank you for your comments and suggestions. In this experiment, gating for the sample tubes was performed by defining the boundary between positive and negative cell populations based on the positive cell population in the single-stained control tubes and the negative cell population in the unstained control tubes. This gating strategy was then applied to the sample tubes, yielding the gating results shown in the figure. In addition, this experiment was conducted using a flow cytometer (SFLO3, Powclin), a novel full-spectrum flow cytometer that employs spectral unmixing to resolve and digitally analyze multiple fluorescence spectra. The coordinate axes in the figure represent only relative fluorescence intensity, and the zero coordinate does not indicate an absolute fluorescence intensity of zero. The use of a relative gating method ensures rigor in the experimental results. In summary, the above experimental results were obtained through analysis using the novel full-spectrum flow cytometer, combined with unstained control tubes and single-stained control tubes for gating and spectral resolution.

Figures including western blot data - all the blot images have been cropped. The full images should at least be included as supplemental data.

Response: Thank you for your comments and suggestions. We appreciate the reviewer's valuable comment concerning the presentation of full blot membranes. In our experimental procedures, all Western blot membranes are sectioned into strips during antibody incubation to optimize incubation efficiency and minimize cross-contamination. The membrane images are included in the supplementary materials to ensure full transparency and validation of our results."

Figures 1 and S1 - the final panel shows a 400x magnification that appears to be most informative. Why was only one panel included at 400x rather than including this for the whole figure?

Response: Thank you for your comments and suggestions. This figure illustrates macrophage activation around the collagen capsule of *T. spiralis* muscle larvae. Since a full histological overview cannot be adequately displayed at 400× magnification, high‑power fields were selectively captured from typical, representative local regions to highlight the key pathological and cellular characteristics.

**Conclusions**
Reviewer #1: Discussion – just reiterates the results in a more summary fashion rather than discussing the results.

Response: Thank you for your comments and suggestions. We sincerely apologize for the inadequate discussion in the initial version. The discussion section has been comprehensively revised, with in-depth analysis of the potential regulatory mechanisms added.

Have the authors speculated what proteins in the ES induce increased expression of S100A4 in macrophages?

Response: We greatly appreciate the reviewer’s professional suggestion. At present, we have not yet identified the specific protein components in ES products. We will verify the key functional molecules in ES products in our future research.

**Editorial and Data Presentation Modifications?**

Reviewer #2: Line 14 - Abstract needs a bit more background as an opening sentence or two. Something like: *T. spiralis* is parasitic worm of humans and animals and is a problem because… (eg infects xx people, causes xxx problems). Then lead into your first sentence with something like: A major hinderance to control is the muscle larva is encased within a collagen capsule that is believed….

Response: Thank you for your comments and suggestions. We have added opening sentence in abstract (Lines 14-16).

Line 112 – needs a linking sentence at the start of this paragraph to connect cells and protein expression. Something like: Down at the protein level a component of the process is potentially the S100A4 calcium binding protein, expressed in various cell types.

Response: Thank you for your comments and suggestions. We have made revisions according to the reviewer’ suggestion (Lines 115-116).

Line123 – reference needed

Response: Thank you for your review and comments. In our prior research, we found that the number of S100A4 positive macrophages was significantly increased, yet these data have not been formally published.

Figure 1: scales bars should be used, not x-magnification (eg 400x)

Response: Thank you for your review and comments. Sorry for not describing clearly. Scale bars are indicated in the legend for Figure 1, with sizes of 200 μm (100×) and 50 μm (400×).

Figure 7A – can you quantify the staining?

Response: Thank you for your valuable comment. In the experimental design of this study, we collected diaphragms from mice in each treatment group for HE staining. Meanwhile, muscle larvae were isolated from mouse carcasses by artificial digestion and quantitatively counted to comprehensively evaluate the therapeutic effect of the treatment. HE staining was mainly used to visually demonstrate the morphology of muscle larvae, collagen capsule, inflammatory cell infiltration, and skeletal muscle damage in *T. spiralis*-infected muscle under different treatment. Quantitative analysis of these indicators was not performed in this study.

Line 432 and 435 – specify what statistically significant is being analysed (eg difference)

Response: Thank you for your valuable comment. Sorry for not describing clearly in this section. We have made revisions according to the reviewer’ suggestions (lines 494-500).

**Summary and General Comments**

Reviewer #1: The manuscript by Meng Ying Gao and colleagues describes the cellular input on the formation of the nurse cell surrounding the parasite *Trichinella spiralis* in the muscle tissue. This is a valuable study, however, as discussed above the methods need extensive editing so that the reader can understand what experiments have been carried out. Overall the manuscript would benefit from more explanation of the cell markers being assessed as too much specialist knowledge is assumed. Similarly, the manuscript would benefit from being edited for English language and grammar.
Response: Thank you for your valuable comment. We have supplemented and refined the experimental methods (including antibody information) as suggested, and corrected grammatical errors throughout the manuscript. The detailed revisions can be found in the revised manuscript.

Line 14 & 62 – not necessary to include *T. spiralis* in brackets here. Just use abbreviated form next time the parasite is mentioned in the text.

Response: Thank you for your review and comments. We have made revisions according to the reviewer’ suggestions.
Line 23 – use of ‘lied’ here – maybe use positioned?

Response: Thank you very much for your valuable comments. We have carefully revised the relevant description in the main text according to your suggestion (line 26).
Line 24 – significant presence – based on what factor/statistical test? Sentence is unclear as to what cells coexpression the markers – I’m assuming these are markers/gene?

Response: Thank you for the valuable comment. We have revised the sentence to clearly indicate that these cells are positive for both CD206 (macrophage marker) and α-SMA (myofibroblast marker) (line 27-28).

Reviewer #2: This work examines the S100A4 involvement in *T. spiralis* infection effectively and in reasonable depth.

Response: Thank you for the valuable comment.

Reviewer #3: The authors of this study explored the mechanism of *T. spiralis* cyst formation from a novel perspective. The application of tissue immunology methods led to the hypothesis that S100a4^+^ macrophages increased in mice infected with *T. spiralis*, and that this increase induced M2 polarization. The M2-like macrophage promoted fibroblasts transition to myofibroblasts by secreting S100A4 and TGF-β1 or directly transformed into myofibroblast-like cells. Subsequent experiments *in vitro* and *in vivo* further demonstrated that the inhibition of S100a4 expression led to a weakening of M2 polarization and a reduction in collagen deposition. In light of these findings, the authors proceeded to combine the S100a4 inhibitor niclosamide with albendazole, with the objective of analyzing the therapeutic effect of the combined drug. The research perspective is distinctive and has practical application value. The writing style employed is generally consistent with established standards, yet it is evident that there are some major issues that need to be further improved to ensure the highest level of clarity and accuracy.
**major issues：**

1. To prove that ES can stimulate the increase in S100A4 expression, it is necessary to directly detect its expression in vitro, rather than measuring the transcriptional level of IL-4 in the tissue. I don't think the results in Figure 2 can support the conclusion in line 288-289.

Response: We greatly appreciate your valuable comments. We apologize for the unclear description of our experimental rationale in the manuscript. Initially, we stimulated macrophages with ES products from *T. spiralis* muscle larvae alone and observed only mild upregulation of Arg1 and S100A4. Previous studies reported that IL‑4 is significantly increased in skeletal muscle during trichinellosis, which was also verified by our qPCR data. We therefore considered that macrophages in infected muscle are regulated by both ES products and the inflammatory microenvironment (e.g. IL‑4). To mimic the *in vivo* microenvironment of *T. spiralis* infection, we used ES products combined with IL‑4 to co‑stimulate macrophages. Macrophage polarization and S100A4 expression were then evaluated by Western blot.

We have revised the relevant content in the main text and provided further explanation and clarification in the discussion section.

2. It is challenging to conclusively confirm that S100a4 is expressed by M2 cells solely based on immunostaining of tissue sections. Additionally, determining whether macrophages have transformed into fibroblasts is also quite difficult. The activation of fibroblasts induced by macrophages or MMT requires a large number of *in vitro* experiments to confirm, and no clear conclusion can be drawn solely from the results of co-localization *in vivo*. I think it is necessary to verify this conclusion through single-cell omics techniques and more in vitro experiments.

Response: We greatly appreciate your valuable comments. Macrophage-induced fibroblast activation and macrophage-to-mesenchymal transition (MMT) indeed require extensive *in vitro* experiments for validation. According to your suggestion, we are currently collecting skeletal muscle tissues at different time points after *T. spiralis* infection and performing single-cell sequencing to verify our conclusions.

3. Does the increased transcription level of IL-4 in muscle tissue result from ES-induced M2 polarization? If so, why was IL-4, a strong stimulant that induces M2 polarization, added in the *in vitro* experiments? What is the role of ES in this culture system? I believe that in the *in vitro* culture system, only ES should be added. The author should have more detailed basis for this experiment.

Response: Thank you for your valuable comments. We initially stimulated macrophages with ES products alone and observed only weak M2 polarization. Previous studies and our qPCR data confirmed that IL‑4 is highly expressed in skeletal muscle during *T. spiralis* infection. Therefore, we used ES combined with IL‑4 to mimic the *in vivo* inflammatory microenvironment, rather than to investigate the effect of ES alone. ES serves as a parasite-derived regulator, while IL‑4 represents the host immune microenvironment. We have clarified the experimental design and rationale in the revised manuscript.

4. In Fig 2, when ES acts alone, it has no significant effect on the expression of Arg1, iNOS, and S100A4. It is imperative to elucidate the mechanisms through which ES can stimulate the increase in S100A4 expression in macrophages.

Response: Thank you for your constructive comment. We agree that ES products alone exerted no significant effect on the expression of Arg1, iNOS, and S100A4 in macrophages. In the *in vivo* infection microenvironment, macrophages are exposed to multiple stimuli rather than ES products alone. Our *in vitro* results suggest that ES may play a role in synergizing with host inflammatory factors such as IL‑4 to upregulate S100A4 expression, rather than acting independently.

We have clarified this synergistic effect and the related rationale in the revised manuscript to better illustrate the potential mechanism of ES in regulating S100A4 expression during *T. spiralis* infection.

5. Figure 3. In the DMEM + PCMs group, what is the reason for the increased expression of α-SMA in fibroblasts by the unactivated macrophages? If ES alone can stimulate the extensive expression of α-SMA, then what is the physiological significance of stimulating the expression of α-SMA in fibroblasts through macrophages? The discussion should include such explanations. In addition, in Figure 3, the WB should ensure uniform internal reference and add fluorescence intensity analysis and gray level analysis.

Response: We greatly appreciate your valuable comments. We apologize for not clarifying these points clearly in the main text.

Previous studies have reported that excretory-secretory (ES) products from *T. spiralis* muscle larvae can directly activate fibroblasts, which was also observed in our immunofluorescence results. We further investigated the effect of macrophages on fibroblast activation. Our data showed that unactivated macrophages had no significant effect on fibroblast activation, whereas macrophages activated by ES plus IL‑4 markedly promoted fibroblast activation.

Therefore, we suggest that *T. spiralis* muscle larvae can activate fibroblasts directly via ES products, and may also indirectly promote fibroblast activation and collagen deposition by regulating the immune microenvironment and activating macrophages. We have supplemented and clarified these explanations in the discussion section.

For the Western blot results in Figure 3, although the internal control bands showed slight inconsistency, the overall trend confirmed that activated macrophages markedly promoted fibroblast activation.

Thank you again for your constructive suggestions and insightful comments.

6. What is the significance of the reduction in collagen? If the reduction of collagen enables more albendazole to enter the cyst, why hasn't the efficiency of the combined treatment significantly increased (Fig 7)? When using albendazole alone, can the insecticidal effect on the cyst stage reach over 80%? Relevant references need to be consulted.

Response: We greatly appreciate your valuable and professional comments.

Although the anthelmintic efficiency of the combined treatment did not show a statistically significant increase in Figure 7, the overall trend was still better than that of albendazole alone. In addition, pathological examination revealed that although the wall of the collagen capsule became thinner, its structural integrity remained intact, which may limit drug entry. We will further explore the underlying causes in future studies to provide a reasonable explanation.

According to previous relevant studies, albendazole (50 mg/kg/d) for 3–7 days at 21–35 dpi resulted in 70%–80% muscle larval reduction in *T. spiralis*-infected mice. In this study, similar albendazole treatment at 21 dpi achieved slightly higher efficacy. Distinct from previous studies, we established a synchronized capsule formation model by eliminating intestinal adults. Meanwhile, the differences may be attributed to the limited number of animals and individual differences.

Thank you again for your constructive suggestions.

**Minor issues：**

1. In Line 188 and 193, the writing of "℃" is incorrect. There should be no space between the number and "℃". Please check the entire text.

Response: Thank you for your comment. The relevant content has been revised and corrected in the main text.

2. In Line201-204, What are the dilution factors of the antibodies?

Response: Thank you for your comment. We have added relevant information in the revised manuscript (Lines 264-268).

3. In Figures 1 and S1, except for the merge images, the fluorescence intensities of the other images are too weak. The brightness of the images should be appropriately adjusted.

Response: Thank you for your comment. We have appropriately optimized the image brightness as suggested.

4. Line 298 and 300, with p > 0.05, indicate no significant difference. When presenting the results, they should not be mixed with other results.

Response: Thank you for your comment. The relevant content has been revised and corrected in the main text (Lines 362-370).

5. Figure 7 legend: It should be clearly stated how many mice are in each group.
Response: Thank you for your comment. The relevant content has been revised and corrected in the main text (Line 506).
