# Supplementary material for: S100A4 enhances the pro-fibrotic effect of macrophage to promote collagen capsule formation of Trichinella spiralis
Source: PLoS Negl Trop Dis. 2026 Sep 15;20(9):e0014647. doi: 10.1371/journal.pntd.0014647 (PMC13577483; doi:10.1371/journal.pntd.0014647)
Supplement: Supplementary file 3 [file pntd.0014647.s008.docx]

PNTD-D-26-00112R1

S100A4 enhances the pro-fibrotic effect of macrophage to promote collagen capsule formation of *Trichinella spiralis*

**Methods**

Reviewer #5: The study design is generally appropriate for the stated objective, and the main experimental procedures are clearly described. However, some methodological details should be clarified.

-Line 105-107: The classification of macrophages as simply pro-inflammatory M1 and anti-inflammatory M2 is overly simplified. It is suggested to use “M1-like” and “M2-like” macrophages, or “classically activated M1 macrophages” and “alternatively activated M2 macrophages.”

Response: Thank you very much for your valuable comments. We have revised the manuscript in accordance with your suggestions (at lines 102-107).

-Line 291-293：Change "sealed" to "blocked"，Change "(v/w)" to "(v/v)"，Separate the primary and secondary antibodies.

Response: Thank you very much for your careful review and constructive suggestions. Corresponding revisions have been made in the revised manuscript (Lines 301-309).

**Results**

Reviewer #5: The results are generally presented in a logical order, and the figures support the main findings. However, several descriptions are inconsistent with the statistical results and should be checked.

-Line 358-359: The manuscript states that “The mRNA level of IL-4 was significantly up-regulated in infected muscle from 14 dpi (*P* < 0.01).” However, the corresponding comparison in S2 Fig is marked as “ns.” Please check the statistical results and ensure that the description in the text is consistent with the figure.

Response: Thank you very much for your careful review. After thorough re-examination of S2 Fig, we confirm that mRNA level of *Il-4* was significantly upregulated in infected muscle at 14 dpi (*P* < 0.01), followed by a gradual decrease. We have revised the description in the manuscript (Lines 369-370).

-Line 438–439: The manuscript states that “inhibition of S100A4 could reduce the expression of α-SMA (*P* > 0.05 at 21 and 28 dpi).” Since the differences at both time points were not statistically significant, the conclusion that S100A4 inhibition reduced α-SMA expression is not supported.

Response: Thank you for this careful and rigorous reminder. We acknowledge that the original sentence was ambiguous and misleading. We have rewritten this description in Lines 456-458.

-Line 465: The title of Fig 6 states that niclosamide attenuated TGF-β signaling. However, only TGF-β1 expression appears to have been measured. It would be more accurate to use “reduced TGF-β1 expression” unless downstream signaling molecules were also examined.

Response: Thank you for this valuable correction. We admit the original figure title was overly generalized. Since only TGF-β1 expression was measured without detecting downstream signaling components, we have revised the relevant content (Lines 485-486).

**Conclusions**

Reviewer #5: The main conclusions are generally consistent with the results. Some statements should be moderated. For example, “directly transdifferentiated into myofibroblasts” may be revised to “acquired myofibroblast-like features.” The statement “developed a new therapeutic strategy” may be revised to “may provide a potential adjunctive strategy.”

Response: Sincerely thank you for your precious suggestions and revisions. We have revised the manuscript strictly in accordance with your suggestions (Lines 45-48).

**Editorial and Data Presentation Modifications?**

Reviewer #5: The manuscript is generally well presented. The following issues should be addressed to improve its clarity and accuracy.

-Line 58: change "macrophage" to "macrophages"

Response: Sincerely thank you for your precious suggestions. We have revised the manuscript strictly in accordance with your suggestions (Line 57).

-Line 57-60: Please revise this sentence to improve its grammar and clarity.

Response: Thank you for this valuable correction. We have revised the manuscript to correct grammar and improve textual clarity in accordance with your suggestions (Lines 56-60).

-Line 388: change “could active fibroblasts” to “could activate fibroblasts”

Response: Thank you for this valuable correction; corresponding revisions have been made in the manuscript (Line 406).

-Line 395: change “bolt" to "blot”

Response: Thank you for this valuable correction; corresponding revisions have been made in the manuscript (Line 413).

-Line 525: change “the co-expressions of” to “the co-expression of”

Response: Sincerely thank you for your precious suggestions. We have revised the manuscript strictly in accordance with your suggestions (Line 544).

-Line 564: change “Studies have showed” to “Studies have shown,”

Response: Sincerely thank you for your precious suggestions. We have revised the manuscript strictly in accordance with your suggestions (Line 583).

-Line 588: change “could attenuates” to “could attenuate.”

Response: Sincerely thank you for your precious suggestions. We have revised the manuscript strictly in accordance with your suggestions (Line 607).

**Summary and General Comments**

Reviewer #4: The study by Gao et al. investigated the effects of *T. spiralis* infection on S100A4 protein expression in muscle cells and its potential consequences on macrophage polarization, transition to myofibroblasts, and collagen deposition. The authors also performed parasiticidal testing of the S100A4 transcriptional inhibitor, niclosamide, in comparison with albendazole, to further explore the potential translational application of their findings. Overall, the study design and experiments were rigorous, and I appreciate the authors’ effort in conducting this work. Most of my concerns relate to the English usage and several statements that appear to overstate the findings. My comments are listed below:

1. Comments on English usage

1.1 Use of abbreviations

Line 104: The manuscript mentioned the M2 type of macrophage for the first time, but only later explained on Lines 107 that M2 macrophages refer to anti-inflammatory macrophages. Please define the term at its first appearance.

Response: Thank you for the valuable suggestions. As suggested, we supplemented full definitions for M1-like and M2-like macrophage phenotypes at their first appearance in the text (102-107).

Extracellular matrix (ECM) was abbreviated on Line 110, but the full term was used again on Lines 541 and 625. Please make the usage consistent throughout the manuscript.

Response: Many thanks for pointing out this inconsistent terminology. We have revised all relevant positions. From the first definition onwards, we uniformly use the abbreviation ECM without repeating the full term to achieve consistent writing norms.

Albendazole was written in full throughout most of the manuscript, but the abbreviation started being used from Line 603 onward. Please use either the full term or abbreviation consistently after the first definition.

Response: We are truly grateful to the reviewer for carefully spotting the inconsistent use of terminology in our text. We have unified the expression of albendazole throughout the manuscript (Lines 621-628).

1.2 The manuscript sometimes refers to the same concept using different terms, especially in the Materials and Methods section, which may confuse readers. For example:

“Medium supplemented with 10% fetal bovine serum” (Line 150) and “complete medium” (Line 157)

Response: Thank you for the helpful suggestion. We have standardized and unified all terminology across the manuscript to maintain consistent expression.

“Peritoneal macrophage” (Line 173) and “Peritoneal exudate cells” (Line 186)

Response: We sincerely appreciate the reviewer’s careful reading and valuable comment. There is an essential experimental distinction between the two terms: peritoneal exudate cells are the unfractionated total peritoneal cell mixture, while peritoneal macrophages are purified adherent cells obtained only after adherent screening. We collected peritoneal exudate cells during the experiment, which were purified via adherent culture before being subjected to subsequent assays.

1.3 Unclear statements

Lines 92–93: “Some workers have observed capsule formation in animals…”

I am not sure whether the term “workers” is appropriate in this context. Terms such as “researchers,” “studies,” or “previous reports” may be more suitable.

Response: Thank you for this valuable suggestion. We recognize that “workers” is an outdated and informal expression in scientific papers. Accordingly, we changed the term to “previous researchers” in the revised manuscript (Line 92).

Line 131: The manuscript mentioned α-SMA^+^ myofibroblasts without prior explanation of α-SMA. Readers unfamiliar with this marker may not understand its significance. If the authors wish to keep this sentence, additional background information should be provided. In my opinion, this sentence could also be removed, as it is not a major finding of the study and is not strongly connected to the main context.

Response: We appreciate this constructive suggestion. Considering that this description is not a core finding of our research and weakly linked to the main theme, we have deleted this sentence from the revised manuscript (Line 131).

1.4 Other language comments

There is no need to capitalize “W” in western blot as it is a common term, unlike “Southern blot,” which was named after its inventor.

Response: Thank you for this careful correction. We have uniformly changed “Western blot” to lowercase “western blot” throughout the manuscript in accordance with this nomenclature rule.

The study included multiple experiments with different treatment conditions, but the current writing style makes it difficult to follow how many treatment groups were included in each experiment. In addition, only the subsection “Therapeutic effect of albendazole and niclosamide combination on T. spiralis-infected mice” explicitly defined the treatment groups as G1, G2, G3, and G4. Please revise this part to improve clarity and maintain consistency throughout the manuscript.

For the English usage, I would suggest professional English editing for the manuscript, particularly because the study design itself is already quite complex, especially in the Materials and Methods section. Improving the language clarity would greatly help readers better follow and understand the study.

Response: Thank you for your constructive suggestions. We have revised all relevant sections to clearly specify the number of treatment groups in each experiment and adopted consistent unified standardized uniform group labels throughout the manuscript to eliminate inconsistent descriptions.

In addition, we arrange professional English language editing for the entire manuscript, with special attention paid to the complicated Materials and Methods section to improve readability.

2. Overstatement Issues

In the Results section, some concluding sentences at the end of the subsections appear overstated or potentially misleading. Please see the details below.

Lines 385–387:

“Overall, these results confirmed that S100A4 was mainly expressed in M2 macrophages and could be induced by IL-4 with ES of *T. spiralis* ML addition.”

The results showed that the percentage of M2 macrophages significantly increased in both the IL-4 and IL-4+ES treatment groups, which was likely mainly driven by IL-4 itself. However, the current wording may lead readers to interpret that only the IL-4+ES condition induced M2 macrophage polarization. Please revise this statement to better reflect the findings.

Response: We appreciate this valuable comment. We have revised the corresponding paragraph to clearly illustrate that M2 macrophage proportions were significantly elevated in both the IL‑4‑only group and the IL‑4 plus ES groups, and clarified that IL‑4 rather than ES products serves as the primary inducer of M2 polarization, with ES showing no additive promoting effect. The modified text is shown in lines 400-405.

Lines 463–465:

“The results showed that niclosamide could diminish macrophage alternative polarization by inhibiting *S100a4* transcription.”

Inhibition of *S100a4* transcription may be one of the mechanisms of niclosamide, but the drug could also exert additional effects on macrophages through other pathways. Although this limitation was mentioned later in the Discussion section, the current statement in the Results section appears somewhat overstated. Please revise the wording to avoid implying a direct causal mechanism that was not fully demonstrated.

Response: We appreciate this valuable comment. We fully agree that the original sentence implied a direct, exclusive mechanistic link between niclosamide-mediated inhibition of *S100a4* transcription and suppressed alternative macrophage polarization, which was not fully validated in our current study. Accordingly, we have revised the statement to adopt more cautious wording to avoid overinterpreting the causal relationship, and the modified sentence at lines 434-436.

Lines 609–612:

“The combined treatment of ABZ and niclosamide showed a tendency to have better therapeutic efficacy than the single drugs. However, the efficiency of the combined treatment did not show a statistically significant increase.”

The worm burden reduction for ABZ alone and ABZ + niclosamide combination treatment was 81.8% and 84.1%, respectively. Considering the relatively small difference (2.3%) and the lack of statistical significance, it may be overstated to suggest improved therapeutic efficacy of the combined treatment. Please revise this statement accordingly.

Response: We appreciate your valuable critical comment. We have fully revised the corresponding text to cautiously interpret this slight numerical disparity, and avoid overstating the therapeutic advantages of the combination regimen (at lines 628-630).

3. Lines 78–79: “protects the parasite from being damaged by the host’s immune system and anti-*Trichinella* drugs.”

This statement may not be fully accurate. Although the capsule helps protect the muscle larvae (ML) from the host immune response, anti-Trichinella drugs can still penetrate the capsule and exert parasiticidal effects. For example, albendazole is clinically used for treatment of *Trichinella* infection. Therefore, this sentence should be revised to avoid overstating the protective role of the capsule against drug treatment.

Response: We appreciate reviewer’s valuable comment. Accordingly, we have revised the relevant sentence (at lines 78-79).

4. As peritoneal macrophages were used throughout the study, the manuscript should provide information regarding the efficiency and purity of the macrophage isolation protocol. How did the authors confirm that the isolated cells were predominantly macrophages with minimal contamination from other cell types?

Response: We sincerely acknowledge your rigorous reminder. Published protocols cited in the manuscript validate that high-purity macrophages are harvested by plating peritoneal cells and enriching adherent populations following a 4 h incubation. The adherent peritoneal macrophages employed in the present study underwent flow cytometric analysis. The results displayed in S3 Fig. Flow cytometry demonstrated that macrophages constituted more than 90% of the isolated cells.

5. I am curious about the rationale for using different markers of macrophage polarization across experiments. For example, in western blot analysis, iNOS and Arg-1 were used as M1 and M2 markers, respectively, whereas in flow cytometry, CD86 and CD206 were used as the M1 and M2 markers. Please clarify the reason for selecting different markers in different experimental settings.

Response: We thank the reviewer’s insightful comment. The distinct marker sets stem from differences in protein subcellular localization and technical detection principles. iNOS and Arg1 are canonical intracellular functional markers detectable via western blot following cell lysis, whereas CD86 and CD206 are surface antigens suited for live-cell flow cytometry. Relevant details have been supplemented in the Materials and Methods section, as shown in lines 252-254, 264-270.

6. In Fig. 3B, the manuscript stated that the level of α-SMA increased following treatment with IL4+ES-stimulated macrophages. However, the difference in the western blot image was not very clear to me. Please consider quantifying the band intensities and providing statistical analysis to support this conclusion. In addition, the GAPDH band in the ES treatment group appeared noticeably weaker than those in the other groups. Please explain this observation.

Response: Thank you for your rigorous and professional comments. We repeated western blot assays numerous times and found that macrophage co-stimulation with IL-4 and ES slightly upregulated α-SMA expression in fibroblasts. This observation is supported by our immunofluorescence data and our team’s previous studies. Unfortunately, other replicate images displayed unexpected technical artifacts caused by operational errors. Though these flaws do not alter our core conclusion, their presentation quality was unsatisfactory. After comprehensive comparison, we selected the most intact blot shown in the manuscript. GAPDH bands remained consistent across all replicate experiments; the weaker internal control signal in the ES group in this figure stems from unequal protein loading during sample preparation.

To report our findings more rigorously, we have revised the manuscript text. The definitive statement “α-SMA expression was elevated” has been adjusted to “a mild upward trend in α-SMA expression was detected in fibroblasts co-cultured with IL-4+ES-stimulated macrophages”. In our future research, we will standardize all experimental procedures, include more independent sample batches with reliable reproducibility, and conduct comprehensive quantitative statistical analyses to further validate this phenotype (at lines 413-415).

Another major drawback of this study is that the findings were not entirely convincing in demonstrating a strong parasite-specific effect of *T. spiralis* infection. Most of the observed changes were limited to altered expression levels of proteins such as IL-4 and S100A4. Although the authors successfully demonstrated the transition of macrophages into myofibroblasts, the downstream findings appeared to reflect broader immunological responses rather than mechanisms clearly driven by the parasite itself.

In particular, the proposed mechanism seemed to be influenced more strongly by IL-4 stimulation than by the ES products of *T. spiralis*. In addition, niclosamide showed only limited effects on worm burden, which further reduced the impact of the proposed therapeutic implication of targeting this pathway.

Nevertheless, the study still contributes useful information to the field and may provide a basis for future mechanistic investigations.

Response: We sincerely appreciate your detailed and objective comments, and we fully recognize the key limitations pointed out. *T. spiralis* infection upregulates IL-4 to establish a type 2 immune, while parasite-derived ES products act as pathogenic mediators to exacerbate fibrotic injury; together they synergistically induce myofibroblast activation. We have revised the inappropriate descriptions in the original manuscript that downplayed the role of IL-4 while overemphasizing the effect of ES. Meanwhile, we have objectively acknowledged the limited anthelmintic efficacy of niclosamide and redefined its application value: it serves as an adjuvant agent against parasite-induced tissue fibrosis, rather than a potent deworming drug.

Reviewer #5: This manuscript investigates the role of macrophages and S100A4 in collagen capsule formation around *Trichinella spiralis* muscle larvae. The topic is interesting, and the results provide useful information for understanding the interaction between macrophages, fibroblasts, and muscle larvae. The study is generally well designed, and the main conclusions are supported by the data. However, there are many grammatical errors and unclear expressions throughout the manuscript. The English should be carefully revised. After these issues are addressed, the manuscript may be suitable for publication.

Response: We greatly appreciate the reviewer’s valuable suggestions. In response to your comments, we have thoroughly revised the entire manuscript to correct grammatical errors and ambiguous expressions.
