# Supplementary material for: S100A4 enhances the pro-fibrotic effect of macrophage to promote collagen capsule formation of Trichinella spiralis
Source: PLoS Negl Trop Dis. 2026 Sep 15;20(9):e0014647. doi: 10.1371/journal.pntd.0014647 (PMC13577483; doi:10.1371/journal.pntd.0014647)
Supplement: Peer review history

# Peer review: Decision Letter 0

**DOI:** 10.1371/journal.pntd.0014647.r001

**Authors:** Maria Victoria Periago, Robert Adamu SHEY

10 Mar 2026

S100A4 enhances the pro-fibrotic effect of macrophage to promote collagen capsule formation of Trichinella spiralis

PLOS Neglected Tropical Diseases

Dear Dr. Zhang,

Thank you for submitting your manuscript to PLOS Neglected Tropical Diseases. After careful consideration, we feel that it has merit but does not fully meet PLOS Neglected Tropical Diseases's publication criteria as it currently stands. Therefore, we invite you to submit a revised version of the manuscript that addresses the points raised during the review process.

Please submit your revised manuscript within by May 09 2026 11:59PM. If you will need more time than this to complete your revisions, please reply to this message or contact the journal office at plosntds@plos.org. When you're ready to submit your revision, log on to https://www.editorialmanager.com/pntd/ and select the 'Submissions Needing Revision' folder to locate your manuscript file.

Please include the following items when submitting your revised manuscript:

* A letter that responds to each point raised by the editor and reviewer(s). You should upload this letter as a separate file labeled 'Response to Reviewers'. This file does not need to include responses to any formatting updates and technical items listed in the 'Journal Requirements' section below.

* A marked-up copy of your manuscript that highlights changes made to the original version. You should upload this as a separate file labeled 'Revised Manuscript with Track Changes'.

* An unmarked version of your revised paper without tracked changes. You should upload this as a separate file labeled 'Manuscript'.

If you would like to make changes to your financial disclosure, competing interests statement, or data availability statement, please make these updates within the submission form at the time of resubmission. Guidelines for resubmitting your figure files are available below the reviewer comments at the end of this letter.

We look forward to receiving your revised manuscript.

Kind regards,

Robert Adamu SHEY, Ph.D.

Guest Editor

PLOS Neglected Tropical Diseases

María Periago

Section Editor

PLOS Neglected Tropical Diseases

Shaden Kamhawi

co-Editor-in-Chief

PLOS Neglected Tropical Diseases

orcid.org/0000-0003-4304-636XX

Paul Brindley

co-Editor-in-Chief

PLOS Neglected Tropical Diseases

orcid.org/0000-0003-1765-0002

**Journal Requirements:**

1) Please ensure that the CRediT author contributions listed for every co-author are completed accurately and in full.

At this stage, the following Authors/Authors require contributions: Meng Ying Gao, Chen Chen Dong, Yue Chun Tang, Ruo Qi Wang, Li Wang, You Jia Ge, Qiao Yu Geng, Peng Jiang, and Zi Fang Zhang. Please ensure that the full contributions of each author are acknowledged in the "Add/Edit/Remove Authors" section of our submission form.

The list of CRediT author contributions may be found here: https://journals.plos.org/plosntds/s/authorship#loc-author-contributions

2) Please amend your detailed Financial Disclosure statement. This is published with the article. It must therefore be completed in full sentences and contain the exact wording you wish to be published.

- State the initials, alongside each funding source, of each author to receive each grant. For example: "This work was supported by the National Institutes of Health (####### to AM; ###### to CJ) and the National Science Foundation (###### to AM)."

- State what role the funders took in the study. If the funders had no role in your study, please state: "The funders had no role in study design, data collection and analysis, decision to publish, or preparation of the manuscript.".

If you did not receive any funding for this study, please simply state: u201cThe authors received no specific funding for this work.u201d

**Reviewers' Comments:**

Reviewer's Responses to Questions

**Key Review Criteria Required for Acceptance?**

As you describe the new analyses required for acceptance, please consider the following:

**Methods**

-Are the objectives of the study clearly articulated with a clear testable hypothesis stated?

-Is the study design appropriate to address the stated objectives?

-Is the population clearly described and appropriate for the hypothesis being tested?

-Is the sample size sufficient to ensure adequate power to address the hypothesis being tested?

-Were correct statistical analysis used to support conclusions?

-Are there concerns about ethical or regulatory requirements being met?

Reviewer #1: All the method sections need more detail including but not only the suppliers used for the consumables, the conditions used and numbers of animals per group. Methods should be written so that the reader can recreate the protocol if needed. Some detail below -but every section needs editing.

Line 136 – why only female mice?

Line 139 – include ethics number details

Section at line 146 - what temperature were the ML cultured at? How many ML in what volume?

Section at line 156 -number of mice in each group? ML used for infections - were these the ML that were cultured or just recovered from the muscle tissue and washed?

Section at line 163 – peritoneal cavity of which mice? Peritoneal macrophages were those stuck to the plate? More detail needed. Supplier details needed for IL-4. Details for cell stimulation – what temperature? Details for Western blot.

Section at line 173 – details of culture, immunofluorescence and Western blot.

Section at line 181 – specific details for RNA extraction and qPCR including supplier names, gene accession numbers

Section at line 187 – details needed

Reviewer #2: adequate

Reviewer #3: yes

**********

**Results**

-Does the analysis presented match the analysis plan?

-Are the results clearly and completely presented?

-Are the figures (Tables, Images) of sufficient quality for clarity?

Reviewer #1: The results are difficult to follow at times, particularly as enough information hasn't been provided regarding the cell markers being used, specifically for non-immunology specialists that may be reading this manuscript.

Line 258 – what is F4/80?

Line 292 – why was only IL4 analysed?

Line 393 – Masson

Figure S3 – how have the CD206 and F4/80 panels been gated? The ES+IL4 and IL4 panels need further confidence.

Figures including western blot data - all the blot images have been cropped. The full images should at least be included as supplemental data.

Figures 1 and S1 - the final panel shows a 400x magnification that appears to be most informative. Why was only one panel included at 400x rather than including this for the whole figure?

Reviewer #2: adequate

Reviewer #3: (No Response)

**********

**Conclusions**

-Are the conclusions supported by the data presented?

-Are the limitations of analysis clearly described?

-Do the authors discuss how these data can be helpful to advance our understanding of the topic under study?

-Is public health relevance addressed?

Reviewer #1: Discussion – just reiterates the results in a more summary fashion rather than discussing the results.

Have the authors speculated what proteins in the ES induce increased expression of S100A4 in macrophages?

Reviewer #2: adequate

Reviewer #3: (No Response)

**********

**Editorial and Data Presentation Modifications?**

Use this section for editorial suggestions as well as relatively minor modifications of existing data that would enhance clarity. If the only modifications needed are minor and/or editorial, you may wish to recommend “Minor Revision” or “Accept”.

Reviewer #1: (No Response)

Reviewer #2: Line 14 - Abstract needs a bit more background as an opening sentence or two. Something like: T. spiralis is parasitic worm of humans and animals and is a problem because… (eg infects xx people, causes xxx problems). Then lead into your first sentence with something like: A major hinderance to control is the muscle larva is encased within a collagen capsule that is believed….

Line 112 – needs a linking sentence at the start of this paragraph to connect cells and protein expression. Something like: Down at the protein level a component of the process is potentially the S100A4 calcium binding protein, expressed in various cell types.

Line123 – reference needed

Figure 1: scales bars should be used, not x-magnification (eg 400x)

Figure 7A – can you quantify the staining?

Line 432 and 435 – specify what statistically significant is being analysed (eg difference)

Reviewer #3: (No Response)

**********

**Summary and General Comments**

Use this section to provide overall comments, discuss strengths/weaknesses of the study, novelty, significance, general execution and scholarship. You may also include additional comments for the author, including concerns about dual publication, research ethics, or publication ethics. If requesting major revision, please articulate the new experiments that are needed.

Reviewer #1: The manuscript by Meng Ying Gao and colleagues describes the cellular input on the formation of the nurse cell surrounding the parasite Trichinella spiralis in the muscle tissue. This is a valuable study, however, as discussed above the methods need extensive editing so that the reader can understand what experiments have been carried out. Overall the manuscript would benefit from more explanation of the cell markers being assessed as too much specialist knowledge is assumed. Similarly the manuscript would benefit from being edited for English language and grammar.

Line 14 & 62 – not necessary to include T. spiralis in brackets here. Just use abbreviated form next time the parasite is mentioned in the text.

Line 23 – use of ‘lied’ here – maybe use positioned?

Line 24 – significant presence – based on what factor/statistical test? Sentence is unclear as to what cells coexpression the markers – I’m assuming these are markers/gene?

Reviewer #2: This work examines the S100A4 involvement in T. spiralis infection effectively and in reasonable depth.

Reviewer #3: The authors of this study explored the mechanism of T. spiralis cyst formation from a novel perspective. The application of tissue immunology methods led to the hypothesis that S100a4+ macrophages increased in mice infected with T. spiralis, and that this increase induced M2 polarization. The M2-like macrophage promoted fibroblasts transition to myofibroblasts by secreting S100A4 and TGF-β1 or directly transformed into myofibroblast-like cells. Subsequent experiments in vitro and in vivo further demonstrated that the inhibition of S100a4 expression led to a weakening of M2 polarization and a reduction in collagen deposition. In light of these findings, the authors proceeded to combine the S100a4 inhibitor niclosamide with albendazole, with the objective of analyzing the therapeutic effect of the combined drug. The research perspective is distinctive and has practical application value. The writing style employed is generally consistent with established standards, yet it is evident that there are some major issues that need to be further improved to ensure the highest level of clarity and accuracy.

major issues:

1. To prove that ES can stimulate the increase in S100A4 expression, it is necessary to directly detect its expression in vitro, rather than measuring the transcriptional level of IL-4 in the tissue. I don't think the results in Figure 2 can support the conclusion in line 288-289.

2. It is challenging to conclusively confirm that S100a4 is expressed by M2 cells solely based on immunostaining of tissue sections. Additionally, determining whether macrophages have transformed into fibroblasts is also quite difficult. The activation of fibroblasts induced by macrophages or MMT requires a large number of in vitro experiments to confirm, and no clear conclusion can be drawn solely from the results of co-localization in vivo. I think it is necessary to verify this conclusion through single-cell omics techniques and more in vitro experiments.

3. Does the increased transcription level of IL-4 in muscle tissue result from ES-induced M2 polarization? If so, why was IL-4, a strong stimulant that induces M2 polarization, added in the in vitro experiments? What is the role of ES in this culture system? I believe that in the in vitro culture system, only ES should be added. The author should have more detailed basis for this experiment.

4. In Fig 2, when ES acts alone, it has no significant effect on the expression of Arg1, iNOS, and S100A4. It is imperative to elucidate the mechanisms through which ES can stimulate the increase in S100A4 expression in macrophages.

5. Figure 3. In the DMEM + PCMs group, what is the reason for the increased expression of α-SMA in fibroblasts by the unactivated macrophages? If ES alone can stimulate the extensive expression of α-SMA, then what is the physiological significance of stimulating the expression of α-SMA in fibroblasts through macrophages? The discussion should include such explanations. In addition, in Figure 3, the WB should ensure uniform internal reference and add fluorescence intensity analysis and gray level analysis.

6. What is the significance of the reduction in collagen? If the reduction of collagen enables more albendazole to enter the cyst, why hasn't the efficiency of the combined treatment significantly increased (Fig 7)? When using albendazole alone, can the insecticidal effect on the cyst stage reach over 80%? Relevant references need to be consulted.

Minor issues:

1. In Line 188 and 193, the writing of "℃" is incorrect. There should be no space between the number and "℃". Please check the entire text.

2. In Line201-204, What are the dilution factors of the antibodies?

3. In Figures 1 and S1, except for the merge images, the fluorescence intensities of the other images are too weak. The brightness of the images should be appropriately adjusted.

4. Line 298 and 300, with p > 0.05, indicate no significant difference. When presenting the results, they should not be mixed with other results.

5. Figure 7 legend: It should be clearly stated how many mice are in each group.

**********

PLOS authors have the option to publish the peer review history of their article (what does this mean?). If published, this will include your full peer review and any attached files.

If you choose “no”, your identity will remain anonymous but your review may still be made public.

**Do you want your identity to be public for this peer review?**  For information about this choice, including consent withdrawal, please see our Privacy Policy.

Reviewer #1: No

Reviewer #2: No

Reviewer #3: No

[NOTE: If reviewer comments were submitted as an attachment file, they will be attached to this email and accessible via the submission site. Please log into your account, locate the manuscript record, and check for the action link "View Attachments". If this link does not appear, there are no attachment files.]

**Figure resubmission:**

**Reproducibility:**

To enhance the reproducibility of your results, we recommend that authors of applicable studies deposit laboratory protocols in protocols.io, where a protocol can be assigned its own identifier (DOI) such that it can be cited independently in the future. Additionally, PLOS ONE offers an option to publish peer-reviewed clinical study protocols. Read more information on sharing protocols at https://plos.org/protocols?utm_medium=editorial-email&utm_source=authorletters&utm_campaign=protocols

# Author comment: Author response to Decision Letter 1

**DOI:** 10.1371/journal.pntd.0014647.r002

7 May 2026

Attachment Submitted filename: Response to Reviewers.docx

# Peer review: Decision Letter 1

**DOI:** 10.1371/journal.pntd.0014647.r003

**Authors:** Maria Victoria Periago, Robert Adamu SHEY

2 Jul 2026

PNTD-D-26-00112R1

S100A4 enhances the pro-fibrotic effect of macrophage to promote collagen capsule formation of Trichinella spiralis

PLOS Neglected Tropical Diseases

Dear Dr. Zhang,

Thank you for submitting your manuscript to PLOS Neglected Tropical Diseases. After careful consideration, we feel that it has merit but does not fully meet PLOS Neglected Tropical Diseases's publication criteria as it currently stands. Therefore, we invite you to submit a revised version of the manuscript that addresses the points raised during the review process.

Please submit your revised manuscript within by Aug 31 2026 11:59PM. If you will need more time than this to complete your revisions, please reply to this message or contact the journal office at plosntds@plos.org. When you're ready to submit your revision, log on to https://www.editorialmanager.com/pntd/ and select the 'Submissions Needing Revision' folder to locate your manuscript file.

Please include the following items when submitting your revised manuscript:

* A letter that responds to each point raised by the editor and reviewer(s). You should upload this letter as a separate file labeled 'Response to Reviewers'. This file does not need to include responses to any formatting updates and technical items listed in the 'Journal Requirements' section below.

* A marked-up copy of your manuscript that highlights changes made to the original version. You should upload this as a separate file labeled 'Revised Manuscript with Track Changes'.

* An unmarked version of your revised paper without tracked changes. You should upload this as a separate file labeled 'Manuscript'.

If you would like to make changes to your financial disclosure, competing interests statement, or data availability statement, please make these updates within the submission form at the time of resubmission. Guidelines for resubmitting your figure files are available below the reviewer comments at the end of this letter.

As the corresponding author, your ORCID iD is verified in the submission system and will appear in the published article. PLOS supports the use of ORCID, and we encourage all coauthors to register for an ORCID iD and use it as well. Please encourage your coauthors to verify their ORCID iD within the submission system before final acceptance, as unverified ORCID iDs will not appear in the published article. Only the individual author can complete the verification step; PLOS staff cannot verify ORCID iDs on behalf of authors.

We look forward to receiving your revised manuscript.

Kind regards,

Robert Adamu SHEY, Ph.D.

Guest Editor

PLOS Neglected Tropical Diseases

Maria Victoria Periago

Section Editor

PLOS Neglected Tropical Diseases

Shaden Kamhawi

co-Editor-in-Chief

PLOS Neglected Tropical Diseases

orcid.org/0000-0003-4304-636XX

Paul Brindley

co-Editor-in-Chief

PLOS Neglected Tropical Diseases

orcid.org/0000-0003-1765-0002

**Additional Editor Comments (if provided):**

**Journal Requirements:**

If the reviewer comments include a recommendation to cite specific previously published works, please review and evaluate these publications to determine whether they are relevant and should be cited. There is no requirement to cite these works unless the editor has indicated otherwise.

1) Please amend your detailed Financial Disclosure statement. This is published with the article. It must therefore be completed in full sentences and contain the exact wording you wish to be published.

i) State the initials, alongside each funding source, of each author to receive each grant. For example: "This work was supported by the National Institutes of Health (####### to AM; ###### to CJ) and the National Science Foundation (###### to AM)."

ii) State what role the funders took in the study. If the funders had no role in your study, please state: "The funders had no role in study design, data collection and analysis, decision to publish, or preparation of the manuscript."

iii) If any authors received a salary from any of your funders, please state which authors and which funders..

If you did not receive any funding for this study, please simply state: u201cThe authors received no specific funding for this work.u201d.

**Reviewers' Comments:**

Reviewer's Responses to Questions

**Key Review Criteria Required for Acceptance?**

As you describe the new analyses required for acceptance, please consider the following:

**Methods**

-Are the objectives of the study clearly articulated with a clear testable hypothesis stated?

-Is the study design appropriate to address the stated objectives?

-Is the population clearly described and appropriate for the hypothesis being tested?

-Is the sample size sufficient to ensure adequate power to address the hypothesis being tested?

-Were correct statistical analysis used to support conclusions?

-Are there concerns about ethical or regulatory requirements being met?

Reviewer #3: (No Response)

Reviewer #4: -Are the objectives of the study clearly articulated with a clear testable hypothesis stated?

Yes

-Is the study design appropriate to address the stated objectives?

Yes

-Is the population clearly described and appropriate for the hypothesis being tested?

Yes

-Is the sample size sufficient to ensure adequate power to address the hypothesis being tested?

Not applicable

-Were correct statistical analysis used to support conclusions?

Yes

-Are there concerns about ethical or regulatory requirements being met?

No

Reviewer #5: The study design is generally appropriate for the stated objective, and the main experimental procedures are clearly described. However, some methodological details should be clarified.

-Line 105-107: The classification of macrophages as simply pro-inflammatory M1 and anti-inflammatory M2 is overly simplified. It is suggested to use “M1-like” and “M2-like” macrophages, or “classically activated M1 macrophages” and “alternatively activated M2 macrophages.”

-Line 291-293:Change "sealed" to "blocked",Change "(v/w)" to "(v/v)",Separate the primary and secondary antibodies.

**********

**Results**

-Does the analysis presented match the analysis plan?

-Are the results clearly and completely presented?

-Are the figures (Tables, Images) of sufficient quality for clarity?

Reviewer #3: (No Response)

Reviewer #4: -Does the analysis presented match the analysis plan?

Yes

-Are the results clearly and completely presented?

See "Summary and General Comments" section

-Are the figures (Tables, Images) of sufficient quality for clarity?

See "Summary and General Comments" section

Reviewer #5: The results are generally presented in a logical order, and the figures support the main findings. However, several descriptions are inconsistent with the statistical results and should be checked.

-Line 358-359: The manuscript states that “The mRNA level of IL-4 was significantly up-regulated in infected muscle from 14 dpi (P < 0.01).”However, the corresponding comparison in S2 Fig is marked as “ns.” Please check the statistical results and ensure that the description in the text is consistent with the figure.

-Line 438–439: The manuscript states that “inhibition of S100A4 could reduce the expression of α-SMA (P > 0.05 at 21 and 28 dpi).” Since the differences at both time points were not statistically significant, the conclusion that S100A4 inhibition reduced α-SMA expression is not supported.

-Line 465: The title of Fig 6 states that niclosamide attenuated TGF-β signaling. However, only TGF-β1 expression appears to have been measured. It would be more accurate to use “reduced TGF-β1 expression” unless downstream signaling molecules were also examined.

**********

**Conclusions**

-Are the conclusions supported by the data presented?

-Are the limitations of analysis clearly described?

-Do the authors discuss how these data can be helpful to advance our understanding of the topic under study?

-Is public health relevance addressed?

Reviewer #3: (No Response)

Reviewer #4: -Are the conclusions supported by the data presented?

See "Summary and General Comments" section

-Are the limitations of analysis clearly described?

Could be improved

-Do the authors discuss how these data can be helpful to advance our understanding of the topic under study?

Yes

-Is public health relevance addressed?

Yes

Reviewer #5: The main conclusions are generally consistent with the results. Some statements should be moderated. For example, “directly transdifferentiated into myofibroblasts” may be revised to “acquired myofibroblast-like features.” The statement “developed a new therapeutic strategy” may be revised to “may provide a potential adjunctive strategy.”

**********

**Editorial and Data Presentation Modifications?**

Use this section for editorial suggestions as well as relatively minor modifications of existing data that would enhance clarity. If the only modifications needed are minor and/or editorial, you may wish to recommend “Minor Revision” or “Accept”.

Reviewer #3: (No Response)

Reviewer #4: -

Reviewer #5: The manuscript is generally well presented. The following issues should be addressed to improve its clarity and accuracy.

-Line 58: change "macrophage" to "macrophages"

-Line 57-60: Please revise this sentence to improve its grammar and clarity.

-Line 388: change “could active fibroblasts” to “could activate fibroblasts”

-Line 395: change “bolt" to "blot”

-Line 525: change“the co-expressions of” to “the co-expression of”

-Line 564: change “Studies have showed” to “Studies have shown,”

-Line 588: change “could attenuates” to “could attenuate.”

**********

**Summary and General Comments**

Use this section to provide overall comments, discuss strengths/weaknesses of the study, novelty, significance, general execution and scholarship. You may also include additional comments for the author, including concerns about dual publication, research ethics, or publication ethics. If requesting major revision, please articulate the new experiments that are needed.

Reviewer #3: (No Response)

Reviewer #4: The study by Gao et al. investigated the effects of T. spiralis infection on S100A4 protein expression in muscle cells and its potential consequences on macrophage polarization, transition to myofibroblasts, and collagen deposition. The authors also performed parasiticidal testing of the S100A4 transcriptional inhibitor, niclosamide, in comparison with albendazole, to further explore the potential translational application of their findings. Overall, the study design and experiments were rigorous, and I appreciate the authors’ effort in conducting this work. Most of my concerns relate to the English usage and several statements that appear to overstate the findings. My comments are listed below:

1. Comments on English usage

1.1 Use of abbreviations

� Line 104: The manuscript mentioned the M2 type of macrophage for the first time, but only later explained on Lines 107 that M2 macrophages refer to anti-inflammatory macrophages. Please define the term at its first appearance.

� Extracellular matrix (ECM) was abbreviated on Line 110, but the full term was used again on Lines 541 and 625. Please make the usage consistent throughout the manuscript.

� Albendazole was written in full throughout most of the manuscript, but the abbreviation started being used from Line 603 onward. Please use either the full term or abbreviation consistently after the first definition.

1.2 The manuscript sometimes refers to the same concept using different terms, especially in the Materials and Methods section, which may confuse readers. For example:

� “Medium supplemented with 10% fetal bovine serum” (Line 150) and “complete medium” (Line 157)

� “Peritoneal macrophage” (Line 173) and “Peritoneal exudate cells” (Line 186)

1.3 Unclear statements

� Lines 92–93: “Some workers have observed capsule formation in animals…”

I am not sure whether the term “workers” is appropriate in this context. Terms such as “researchers,” “studies,” or “previous reports” may be more suitable.

� Line 131: The manuscript mentioned α-SMA+ myofibroblasts without prior explanation of α-SMA. Readers unfamiliar with this marker may not understand its significance. If the authors wish to keep this sentence, additional background information should be provided. In my opinion, this sentence could also be removed, as it is not a major finding of the study and is not strongly connected to the main context.

1.4 Other language comments

� There is no need to capitalize “W” in western blot as it is a common term, unlike “Southern blot,” which was named after its inventor.

� The study included multiple experiments with different treatment conditions, but the current writing style makes it difficult to follow how many treatment groups were included in each experiment. In addition, only the subsection “Therapeutic effect of albendazole and niclosamide combination on T. spiralis-infected mice” explicitly defined the treatment groups as G1, G2, G3, and G4. Please revise this part to improve clarity and maintain consistency throughout the manuscript.

For the English usage, I would suggest professional English editing for the manuscript, particularly because the study design itself is already quite complex, especially in the Materials and Methods section. Improving the language clarity would greatly help readers better follow and understand the study.

2. Overstatement Issues

In the Results section, some concluding sentences at the end of the subsections appear overstated or potentially misleading. Please see the details below.

� Lines 385–387:

“Overall, these results confirmed that S100A4 was mainly expressed in M2 macrophages and could be induced by IL-4 with ES of T. spiralis ML addition.”

The results showed that the percentage of M2 macrophages significantly increased in both the IL-4 and IL-4+ES treatment groups, which was likely mainly driven by IL-4 itself. However, the current wording may lead readers to interpret that only the IL-4+ES condition induced M2 macrophage polarization. Please revise this statement to better reflect the findings.

� Lines 463–465:

“The results showed that niclosamide could diminish macrophage alternative polarization by inhibiting S100a4 transcription.”

Inhibition of S100a4 transcription may be one of the mechanisms of niclosamide, but the drug could also exert additional effects on macrophages through other pathways. Although this limitation was mentioned later in the Discussion section, the current statement in the Results section appears somewhat overstated. Please revise the wording to avoid implying a direct causal mechanism that was not fully demonstrated.

� Lines 609–612:

“The combined treatment of ABZ and niclosamide showed a tendency to have better therapeutic efficacy than the single drugs. However, the efficiency of the combined treatment did not show a statistically significant increase.”

The worm burden reduction for ABZ alone and ABZ + niclosamide combination treatment was 81.8% and 84.1%, respectively. Considering the relatively small difference (2.3%) and the lack of statistical significance, it may be overstated to suggest improved therapeutic efficacy of the combined treatment. Please revise this statement accordingly.

3. Lines 78–79: “protects the parasite from being damaged by the host’s immune system and anti-Trichinella drugs.”

This statement may not be fully accurate. Although the capsule helps protect the muscle larvae (ML) from the host immune response, anti-Trichinella drugs can still penetrate the capsule and exert parasiticidal effects. For example, albendazole is clinically used for treatment of Trichinella infection. Therefore, this sentence should be revised to avoid overstating the protective role of the capsule against drug treatment.

4. As peritoneal macrophages were used throughout the study, the manuscript should provide information regarding the efficiency and purity of the macrophage isolation protocol. How did the authors confirm that the isolated cells were predominantly macrophages with minimal contamination from other cell types?

5. I am curious about the rationale for using different markers of macrophage polarization across experiments. For example, in western blot analysis, iNOS and Arg-2 were used as M1 and M2 markers, respectively, whereas in flow cytometry, CD86 and CD206 were used as the M1 and M2 markers. Please clarify the reason for selecting different markers in different experimental settings.

6. In Fig. 3B, the manuscript stated that the level of α-SMA increased following treatment with IL4+ES-stimulated macrophages. However, the difference in the western blot image was not very clear to me. Please consider quantifying the band intensities and providing statistical analysis to support this conclusion. In addition, the GAPDH band in the ES treatment group appeared noticeably weaker than those in the other groups. Please explain this observation.

Another major drawback of this study is that the findings were not entirely convincing in demonstrating a strong parasite-specific effect of T. spiralis infection. Most of the observed changes were limited to altered expression levels of proteins such as IL-4 and S100A4. Although the authors successfully demonstrated the transition of macrophages into myofibroblasts, the downstream findings appeared to reflect broader immunological responses rather than mechanisms clearly driven by the parasite itself.

In particular, the proposed mechanism seemed to be influenced more strongly by IL-4 stimulation than by the ES products of T. spiralis. In addition, niclosamide showed only limited effects on worm burden, which further reduced the impact of the proposed therapeutic implication of targeting this pathway.

Nevertheless, the study still contributes useful information to the field and may provide a basis for future mechanistic investigations.

Reviewer #5: This manuscript investigates the role of macrophages and S100A4 in collagen capsule formation around Trichinella spiralis muscle larvae. The topic is interesting, and the results provide useful information for understanding the interaction between macrophages, fibroblasts, and muscle larvae. The study is generally well designed, and the main conclusions are supported by the data. However, there are many grammatical errors and unclear expressions throughout the manuscript. The English should be carefully revised. After these issues are addressed, the manuscript may be suitable for publication.

**********

PLOS authors have the option to publish the peer review history of their article (what does this mean?). If published, this will include your full peer review and any attached files.

If you choose “no”, your identity will remain anonymous but your review may still be made public.

**Do you want your identity to be public for this peer review?**  For information about this choice, including consent withdrawal, please see our Privacy Policy.

Reviewer #3: No

Reviewer #4: No

Reviewer #5: No

[NOTE: If reviewer comments were submitted as an attachment file, they will be attached to this email and accessible via the submission site. Please log into your account, locate the manuscript record, and check for the action link "View Attachments". If this link does not appear, there are no attachment files.]

**Figure resubmission:**

**Reproducibility:**

To enhance the reproducibility of your results, we recommend that authors of applicable studies deposit laboratory protocols in protocols.io, where a protocol can be assigned its own identifier (DOI) such that it can be cited independently in the future. Additionally, PLOS ONE offers an option to publish peer-reviewed clinical study protocols. Read more information on sharing protocols at https://plos.org/protocols?utm_medium=editorial-email&utm_source=authorletters&utm_campaign=protocols

# Author comment: Author response to Decision Letter 2

**DOI:** 10.1371/journal.pntd.0014647.r004

21 Jul 2026

Attachment Submitted filename: Response_to_Reviewers_auresp_2.docx

# Editor report: Decision Letter 2

**DOI:** 10.1371/journal.pntd.0014647.r005

**Authors:** Maria Victoria Periago, Robert Adamu SHEY

5 Aug 2026

Dear prof. Zhang,

We are pleased to inform you that your manuscript 'S100A4 enhances the pro-fibrotic effect of macrophage to promote collagen capsule formation of Trichinella spiralis' has been provisionally accepted for publication in PLOS Neglected Tropical Diseases.

Before your manuscript can be formally accepted you will need to complete some formatting changes, which you will receive in a follow up email. A member of our team will be in touch with a set of requests.

Please note that your manuscript will not be scheduled for publication until you have made the required changes, so a swift response is appreciated.

IMPORTANT: The editorial review process is now complete. PLOS will only permit corrections to spelling, formatting or significant scientific errors from this point onwards. Requests for major changes, or any which affect the scientific understanding of your work, will cause delays to the publication date of your manuscript.

Should you, your institution's press office or the journal office choose to press release your paper, you will automatically be opted out of early publication. We ask that you notify us now if you or your institution is planning to press release the article. All press must be co-ordinated with PLOS.

Thank you again for supporting Open Access publishing; we are looking forward to publishing your work in PLOS Neglected Tropical Diseases.

Best regards,

Robert Adamu SHEY, Ph.D.

Guest Editor

PLOS Neglected Tropical Diseases

Maria Victoria Periago

Section Editor

PLOS Neglected Tropical Diseases

Shaden Kamhawi

co-Editor-in-Chief

PLOS Neglected Tropical Diseases

orcid.org/0000-0003-4304-636XX

Paul Brindley

co-Editor-in-Chief

PLOS Neglected Tropical Diseases

orcid.org/0000-0003-1765-0002

***********************************************************

p.p1 {margin: 0.0px 0.0px 0.0px 0.0px; line-height: 16.0px; font: 14.0px Arial; color: #323333; -webkit-text-stroke: #323333}span.s1 {font-kerning: none

# Editor report: Acceptance letter

**DOI:** 10.1371/journal.pntd.0014647.r006

**Authors:** Maria Victoria Periago, Robert Adamu SHEY

Dear prof. Zhang,

We are delighted to inform you that your manuscript, "S100A4 enhances the pro-fibrotic effect of macrophage to promote collagen capsule formation of Trichinella spiralis," has been formally accepted for publication in PLOS Neglected Tropical Diseases.

We have now passed your article onto the PLOS Production Department who will complete the rest of the publication process. All authors will receive a confirmation email upon publication.

The corresponding author will soon be receiving a typeset proof for review, to ensure errors have not been introduced during production. Please review the PDF proof of your manuscript carefully, as this is the last chance to correct any scientific or type-setting errors. Please note that major changes, or those which affect the scientific understanding of the work, will likely cause delays to the publication date of your manuscript. Note: Proofs for Front Matter articles (Editorial, Viewpoint, Symposium, Review, etc...) are generated on a different schedule and may not be made available as quickly.

Soon after your final files are uploaded, the early version of your manuscript will be published online unless you opted out of this process. The date of the early version will be your article's publication date. The final article will be published to the same URL, and all versions of the paper will be accessible to readers.

For Research Articles, you will receive an invoice from PLOS for your publication fee after your manuscript has reached the completed accept phase. If you receive an email requesting payment before acceptance or for any other service, this may be a phishing scheme. Learn how to identify phishing emails and protect your accounts at https://explore.plos.org/phishing.

Thank you again for supporting open-access publishing; we are looking forward to publishing your work in PLOS Neglected Tropical Diseases.

Best regards,

Shaden Kamhawi

co-Editor-in-Chief

PLOS Neglected Tropical Diseases

Paul Brindley

co-Editor-in-Chief

PLOS Neglected Tropical Diseases
